# Imaging and Molecular Annotation of Xenographs and Tumours (IMAXT): High throughput data and analysis infrastructure

**DOI:** 10.1017/S2633903X23000090

**Published:** 2023-04-14

**Authors:** Eduardo A. González-Solares, Ali Dariush, Carlos González-Fernández, Aybüke Küpcü Yoldaş, Alireza Molaeinezhad, Mohammad Al Sa’d, Leigh Smith, Tristan Whitmarsh, Neil Millar, Nicholas Chornay, Ilaria Falciatori, Atefeh Fatemi, Daniel Goodwin, Laura Kuett, Claire M. Mulvey, Marta Páez Ribes, Fatime Qosaj, Andrew Roth, Ignacio Vázquez-García, Spencer S. Watson, Jonas Windhager, Samuel Aparicio, Bernd Bodenmiller, Ed Boyden, Carlos Caldas, Owen Harris, Sohrab P. Shah, Simon Tavaré, CRUK IMAXT Grand Challenge Team, Dario Bressan, Gregory J. Hannon, Nicholas A. Walton

**Affiliations:** 1 Institute of Astronomy, University of Cambridge, Cambridge, United Kingdom; 2 CRUK Cambridge Institute, Li Ka Shing Centre, University of Cambridge, Cambridge, United Kingdom; 3 McGovern Institute, Department of Biological Engineering, Massachusetts Institute of Technology, Cambridge, MA, USA; 4 McGovern Institute, Department of Brain and Cognitive Sciences, Massachusetts Institute of Technology, Cambridge, MA, USA; 5 Department of Quantitative Biomedicine, University of Zurich, Zurich, Switzerland; 6 Institute of Molecular Life Sciences, University of Zurich, Zurich, Switzerland; 7 Department of Computer Science, University of British Columbia, Vancouver, BC, Canada; 8 Herbert and Florence Irving Institute for Cancer Dynamics, Columbia University, New York, NY, USA; 9 Department of Epidemiology and Biostatistics, Memorial Sloan Kettering Cancer Center, New York, NY, USA; 10 Department of Oncology and Ludwig Institute for Cancer Research, University of Lausanne, Lausanne, Switzerland; 11 Department of Pathology and Laboratory Medicine, University of British Columbia, Vancouver, BC, Canada; 12 Howard Hughes Medical Institute, Department of Physics, Harvard University, Cambridge, MA, USA; 13 Howard Hughes Medical Institute, Department of Chemistry and Chemical Biology, Harvard University, Cambridge, MA, USA; 14 Cambridge Breast Unit, Addenbrooke’s Hospital, Cambridge University Hospital NHS Foundation Trust and NIHR Cambridge Biomedical Research Centre, Cambridge, United Kingdom; 15 Súil Interactive, Dublin, Ireland; 16 New York Genome Center, New York, NY, USA

**Keywords:** Data management, data processing and analysis, fluorescence microscopy, imaging mass cytometry

## Abstract

With the aim of producing a 3D representation of tumors, imaging and molecular annotation of xenografts and tumors (IMAXT) uses a large variety of modalities in order to acquire tumor samples and produce a map of every cell in the tumor and its host environment. With the large volume and variety of data produced in the project, we developed automatic data workflows and analysis pipelines. We introduce a research methodology where scientists connect to a cloud environment to perform analysis close to where data are located, instead of bringing data to their local computers. Here, we present the data and analysis infrastructure, discuss the unique computational challenges and describe the analysis chains developed and deployed to generate molecularly annotated tumor models. Registration is achieved by use of a novel technique involving spherical fiducial marks that are visible in all imaging modalities used within IMAXT. The automatic pipelines are highly optimized and allow to obtain processed datasets several times quicker than current solutions narrowing the gap between data acquisition and scientific exploitation.

## Impact Statement

Challenges of the increasing amount of data produced by current and future instrumentation include the need of available pipelines that can process these data routinely in a timely fashion and data access mechanisms that make working with large datasets achievable. This article addresses these two challenges by describing highly parallel fully automated pipelines that perform image stitching, image registration and image segmentation, and designing a data analysis infrastructure where data analysis takes place close to where data are located using on demand remote resources.

## 1. Introduction

Single-cell analysis providing a detailed genomic and proteomic breakdown of tissues is now well established. Hitherto, spatial information has been lost. Recognizing the importance of understanding the detailed environments of tumors, the Cancer Grand Challenge identified a key challenge to map the molecular and cellular tumor microenvironment (https://cancergrandchallenges.org/challenges/3d-tumour-mapping) in order to define new targets for therapy and prognosis. The imaging and molecular annotation of xenografts and tumors (IMAXT) project is adopting an integrated approach to study tumors and their environment by building a 3D representation that can be explored using virtual reality, and show every single fully annotated cell type in the tumor and surroundings.

The project uses a large variety of technologies and instrumental modalities gathering multi-disciplinary expertise from many international groups including sequencing, molecular biology, statistics, medicine, astronomy, and virtual reality experts.

Serial Two-Photon Tomography^(^^1^^)^ (STPT) is the fastest and most high-throughput of the IMAXT data acquisition modalities. It is the only modality capable of processing sample numbers in the range of hundreds of full-size (centimeter-level) tumors, or thousands of biopsies, and provides full 3D models at single-cell resolution. It also serves as the starting point and sectioning step for our deeper analysis pipelines, including imaging mass cytometry^(^^2^^)^ (IMC), and, in the near future, Expansion Sequencing^(^^3^^)^ (ExSeq) and Multiplexed error-robust fluorescence in situ hybridization^(^^4^^)^ (MERFISH) (which are typically performed on frozen sections). These are complemented by single-cell RNA and DNA sequencing.

**Figure 1.**
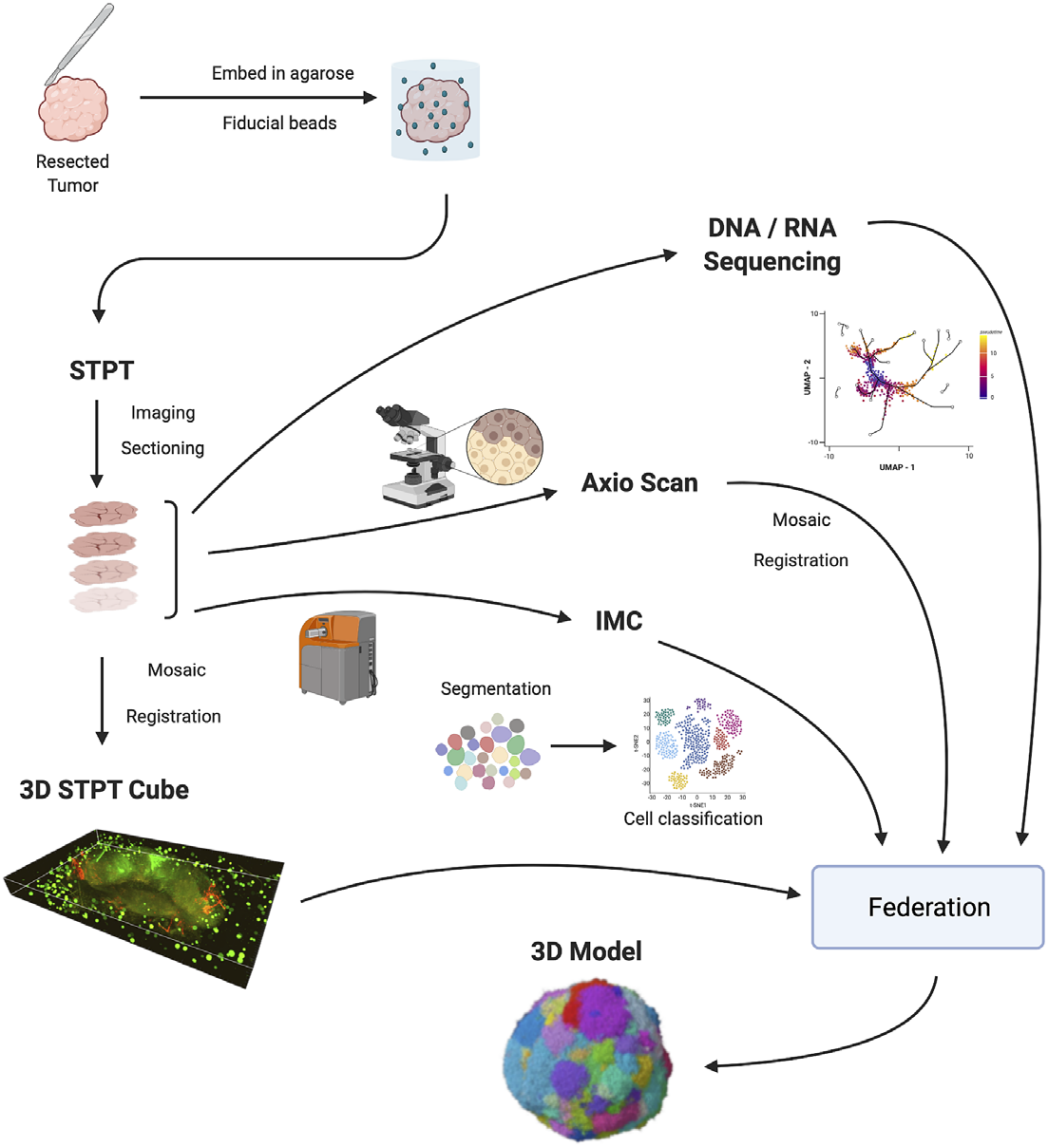
IMAXT pipeline. Once a tumor has been extracted it is embedded in an agarose cube together with spherical beads. The sample is then analyzed in the STPT instrument where it is imaged and cut into thin slices and a multichannel 3D data cube is produced. The slices are then imaged with an Axioscan fluorescence microscope and an IMC mass cytometer. The spherical beads are used for alignment of slices within each sample and for registration between all samples. All imaging is resampled to the STPT reference. All data, including sequencing, is then federated to build an annotated 3D model.

Figure 1 shows the IMAXT data acquisition and analysis workflow. A tumor sample is collected (via biopsy or resection from a mouse implant) and embedded in agarose in order to maintain the sample integrity and allow for sectioning. At the same time, fluorescent spherical agarose beads of about 90 μm in diameter are inserted in the cube in the areas not covered by the sample. These beads will be especially useful during image registration (see Section 4). The size of the final block is around 1 cm^3^. Slices as thin as 15 μm are sectioned using a vibratome of the TissueCyte 2000 instrument (TissueVision Inc., Newton, MA) and imaged through a variety of instruments. STPT performs two-photon fluorescence imaging in four channels a few microns below the surface of the cube. The cube is then cut and each slice is subsequently imaged with fluorescence scanning (Zeiss Axioscan slide scanner) at several wavelengths, allowing for additional fluorescent markers. The slice is then transferred to the IMC device, which provides information on up to 40 individual metal-conjugated antibodies.

Note that there is a bit of abrasion at the cut interface but STPT always images below the surface so the abrasion is not captured. The minor abrasion damage is not an issue for the other modalities as the antibody staining is still effective. Other issues related to deterioration of cell health are not applicable here since there is no live tissue.

Slices are registered using the beads inserted in the agarose cube and all data is resampled to the STPT reference frame. Together with sequencing data, all data is then federated to make an annotated 3D model with each cell having tens to hundreds of descriptors.

Many of these technologies produce large quantities of data that need to be processed before any scientific analysis can be performed. As the technologies mature, rates of data production also increase. As an example, STPT alone generates between 2 and 3 TB of imaging data per sample. A sample can be imaged in about a day. Analysis of these large volumes of data, that are available at high data rates, calls for automatic processing pipelines that can optimally run with high a level of parallelism and produce results in a timely fashion.

IMAXT also presents a number of unique computational challenges, where approaches developed for data handling and image analysis of multi-wavelength survey data in astronomy (e.g., Ref. (5) for initial concepts) have been adapted for use here. In particular, the requirement to register multi-modal image data into a common reference frame to subcellular precision, has led to the development of an image registration technique based on astrometric methods commonly used in astronomy. For instance, large sky surveys require accurate registration and stitching, where the astrometric calibration relies on matching to a well-known defined set of reference marker stars (e.g., stitching thousands of sky images to form a map of the Milky Way’s inner disk^(^^6^^)^, or a multi-epoch, multi-wavelength atlas of the Milky Way’s Bulge^(^^7^^)^). The technique developed here of embedding a “star field” surrounding each tissue sample, allows for efficient and accurate registration across all image data sets, as the embedded “star field” beads are visible in each imaging modality and provide a fixed reference against which positional registration and corrections for image deformations can be made.

From the scientist’s point of view, the traditional scenario where users connect to an archive or repository and download data to their own computers to perform an analysis are bound to be unfeasible. We favor instead a model where users connect to a cloud-based remote system close to where the data are and having available tools to further carry out an analysis using the full power of the “IMAXT cloud.” This model changes the way scientists interact with data, how they solve problems and share results with colleagues. Similar endeavors are also starting across many other scientific fields. Such as the Pangeo project^(^^8^^,^^9^^)^ and the Planetary Computer (https://planetarycomputer.microsoft.com) in earth sciences, and the LSST project in astronomy^(^^10^^)^. In Biology and Biomedical Image Analysis, similar aims are being pursued by, for example, Cytomine (https://cytomine.com) or Renku (https://renkulab.io). There is however no one solution that fits all and here we describe the architecture that best fits our purposes in terms of large data volume analysis, type of datasets to analyze, flexibility, and interoperability between different tools.

The move to cloud-based analyses, however, comes with its own challenges. Analysis pipelines are required which perform tasks with high parallelism, as well as new tools and data formats that allow chunked parallel reads and writes and are cloud storage efficient. In the next sections, we describe our approach to these and other challenges and introduce some of the analysis pipelines and methods used.

This technical report focuses on the data analysis infrastructure and methods used to produce datasets that are ready for further scientific analysis. The pipelines described here generate three-dimensional, molecularly annotated models of breast cancer.

## 2. Results

Efficient processing and analysis of samples acquired by imaging techniques becomes a challenge due to increasingly large data volumes for each modality, increased rate at which these are produced and the variety of modalities that can be used to obtain a complete picture.

In this report, we introduce the infrastructure that we have developed to address these challenges. Data analysis pipelines in the cloud allow for those pipelines to utilize resources as needed and scale when required as they run close to where the data are located. In the same vein, scientists can access the full power of the cloud to perform their analysis and share results with colleagues without having to transfer large amounts of data and with dedicated software tools readily available.

Using this infrastructure we build high throughput automatic pipelines for stitching, registration, and segmentation of the datasets generated by the multiple modalities that are at a later stage federated to build a complex 3D model of a tumor.

Creating a 3D STPT cube from the data obtained by the microscope is a two-step process. In the first instance, we stitch all the fields of view (tiles) that make each slice. In order to do so, we correct the tiles from instrumental effects and compute the offsets between all adjacent tiles using their overlap areas. Using these offsets as free parameters we find the position of each tile in the stage by minimizing the overlap residuals. Once a slice is stitched, we run it through a neural network model trained to segment the beads. The detected beads are then fitted using a high-order Gaussian function to determine the center and diameter. Since the bead diameter is larger than the thickness of the slice, there will be quite a few beads in common between consecutive slices (they will have different diameters but their centers will be accurate). Using these common beads we register consecutive slices pairwise assuming a rigid transformation.

**Figure 2.**
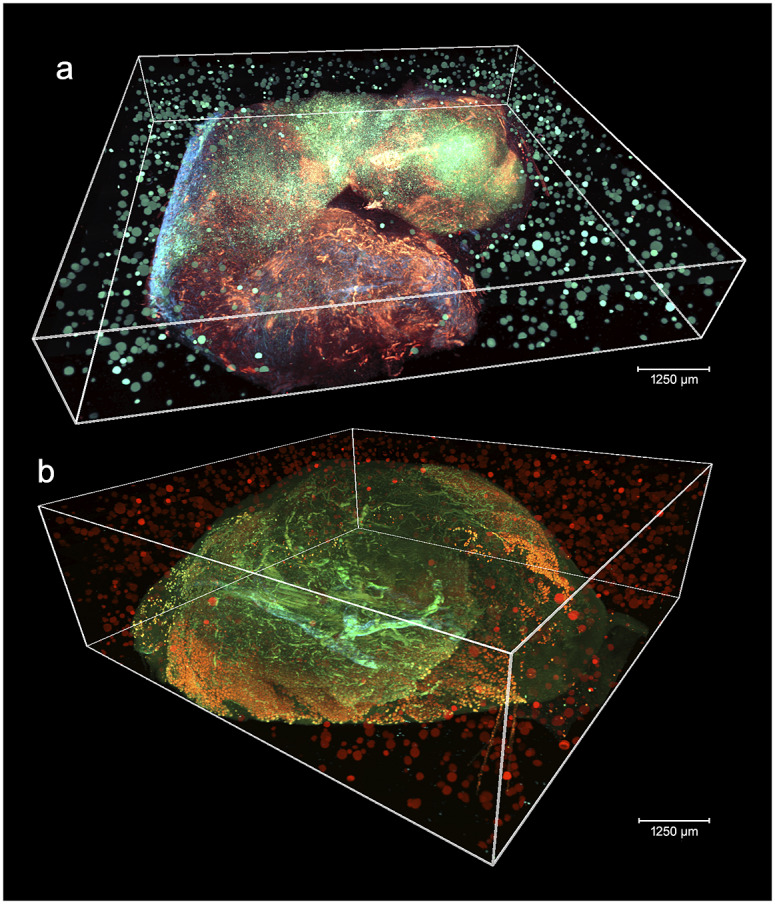
3D visualizations of two stitched STPT cubes with different properties. (a) Volumetric reconstruction from 99 sections of 15 μm This sample was processed by an orthotopic injection of the fluorescent 4 t1-E subclone^(^^11^^)^ shown in channel 3 (green) which is known to undergo vascular mimicry. In addition, this sample was perfused with the DiI lipophilic dye seen in channel 2 (red) to highlight vessel structures. (b) Volumetric reconstruction from 100 sections of 25 μm, each with 5 optical sections of 5 μm from a 4 T1 tumor in a BalbC mouse with the tdTomato marker. The fluorescence beads are clearly visible in the medium outside the biological tissue and prove to be crucial for all stages of registration.

Figure 2 shows two aligned 3D STPT cubes with a different number of physical and optical sections.

The multi-modality registration software is fully compatible with STPT, IMC, and low-magnification fluorescence imaging data (i.e., whole slide scanning). The software has been tested over a sample for which STPT and IMC data have been collected over 20 physical slices, using an Axioscan fluorescence scanning microscope as an intermediate step to simplify slide identification.

We implemented an embedding chemistry based on rigid hydrogels developed by Tissuevision Inc., reaching bead densities up to 10 times higher, and have increased the overlap between the individual image tiles forming the STPT data cube, improving the bead identification. This allowed us to improve the realignment precision on individual (i.e., not belonging to a 3D sample) STPT sections and their matching IMC datasets. Resolutions allowing single-cell realignment are now attainable.

**Figure 3.**
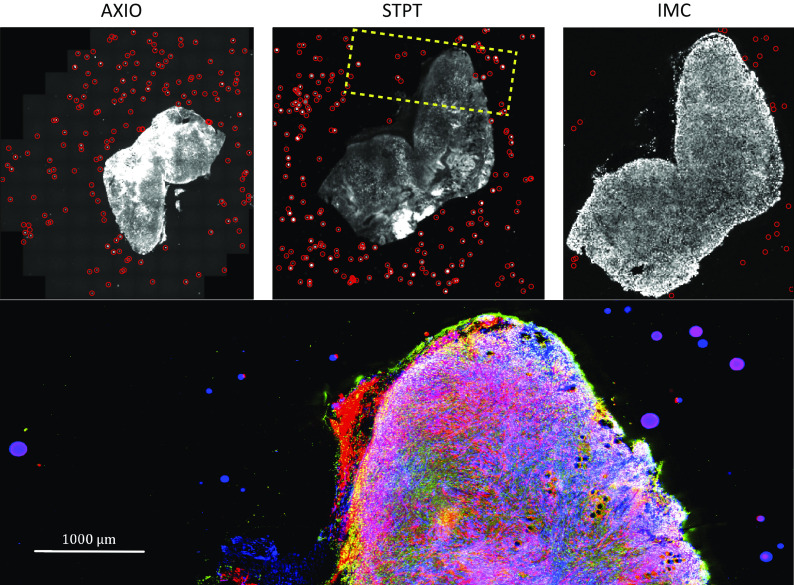
Multi-modality image registration. Registration across different modalities is achieved using the spherical fiducial beads. These are automatically detected on single-channel images, and a measurement of their geometric center is obtained. The top row of images shows the location of detected beads in an Axioscan, STPT, and IMC slide. A first coarse alignment is carried out using 32× downsampled images, and with this and the fiducial center coordinates, an affine transformation matrix is calculated. With this, we can reproject between modalities. The result can be seen in the bottom panel, an inset of the box outlined in the STPT image once registration has finished; here Axioscan occupies the red channel, IMC the green, and STPT the blue.

Computing times for registration between datasets are 2 min per slice per core, and the typical fiducial matching error is of 5 μm between STPT and Axioscan and of 8 μm between IMC and Axioscan. Figure 3 shows the result of the full registration and reprojection.

**Figure 4.**
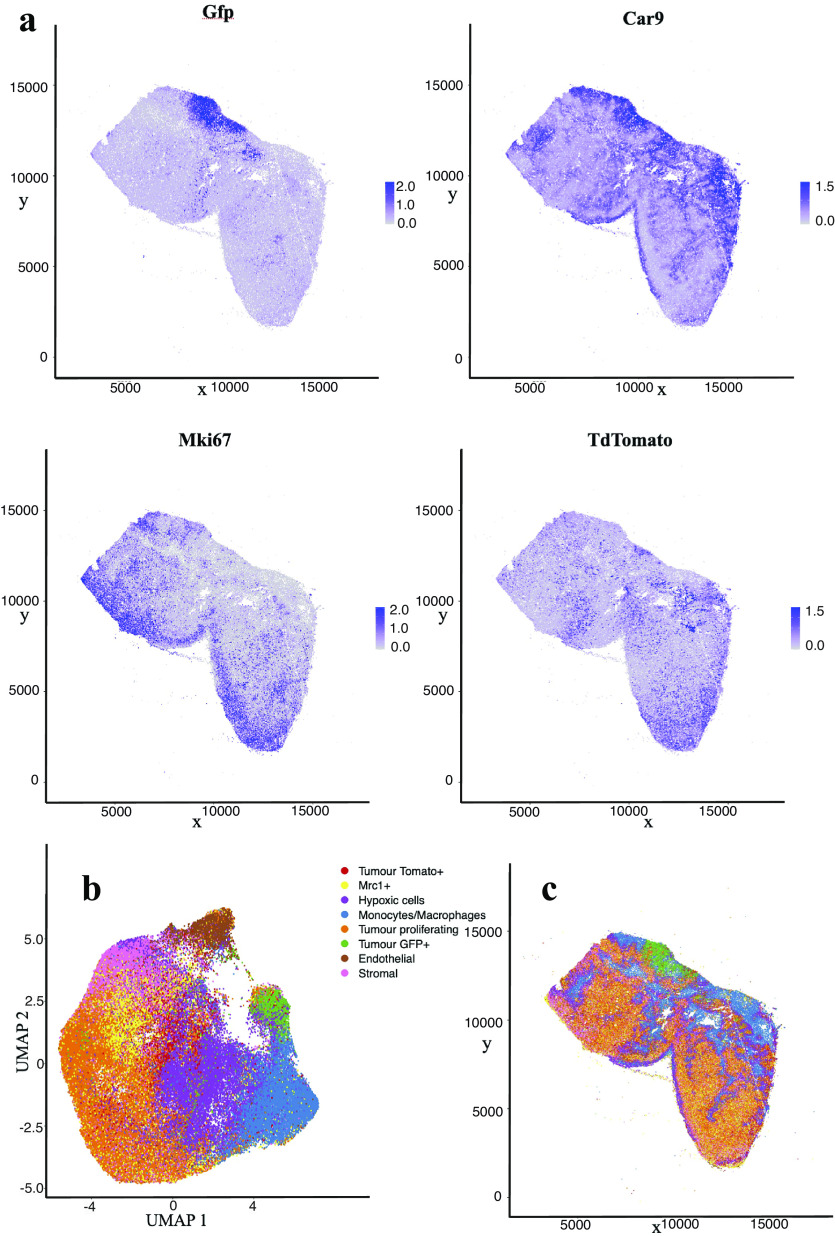
Analysis of IMC data segmentation of the 3D sample. (a) Marker abundance plots for a representative 2D section of the sample. Each dot is a segmented cells and signal intensity corresponds to the normalized abundance of the marker in the cell. We show GFP and TdTomato (tumor cell populations), proliferation (Ki67), and Hypoxia markers (Car9). (b) UMAP dimensional reduction plot for the dataset. (c) Spatial plot of a representative 2D section of the dataset. Each color corresponds to a different cell type predicted by leiden clustering on the data.

The nuclear segmentation pipeline achieves a good performance on IMC images using thresholding-based segmentation. Using the iridium DNA intercalator as a marker of the nuclear channel, the pipeline extracts cell locations and relevant areas in this channel and then measures the integrated intensity over each cell’s segmented area in every channel. Our results show a high correlation of detections when compared to manual count. Some 89% of cells are correctly detected while we estimate a false positive detection rate of 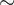 10%. Figure 4 shows the cell distribution and classification obtained from the segmentation results in a representative section of the sample. Results from the IMC segmentation method described here have also been validated scientifically elsewhere^(^^12^^)^.

Our baseline IMAXT infrastructure and analysis system release includes the following instrument-specific pipelines:

- *STPT mosaic pipeline.* Performs stitching of individual tiles for each slice, taking into account overlaps between tiles and geometric distortions followed by 3D registration of all slides in a sample.
- *STPT tissue segmentation pipeline.* A tailored pipeline optimized for segmentation and reconstruction of stroma and vascular structures in STPT datasets.
- *Axioscan mosaic pipeline.* Similar to the STPT pipeline but optimized for the fluorescence scanning datasets.
- *IMC nuclear segmentation.* Performs nuclear segmentation, currently using a watershed segmentation-based algorithm, and produces a catalogue of cell positions, shapes, intensities, and image-derived properties.
- *MERFISH mosaic and decoding.* This pipeline performs stitching, segmentation, and decoding of genes^(^^4^^)^.

We provide a detailed description of the algorithms involved in stitching, registration, nuclear segmentation, volume segmentation, and data federation in Section 4, including the final coregistration of the IMC segmented images into the STPT ground truth reference frame where registration errors of 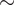 7 μm are achieved (median error across the sample).

## 3. Discussion

New imaging technologies and techniques are opening the possibility to explore fully molecularly annotated tissue samples at the subcellular level. The full discovery potential is realized by successfully federating the multi-pathway input data streams, such that the accurately registered three-dimensional model of the tissue sample can be realized.

IMAXT is taking a holistic approach, in order to meet one of the current Cancer Grand Challenges, generating detailed maps at the single cell level of all cells and cell types in a tumor, creating 3D renderings of tumor models in which both the tumor cells and cells of the tumor microenvironment are annotated using a fluorescent code. The 3D renderings are created from samples placed in a STPT microscope. Alternating optical and physical sections, producing overlapping 3D plates are stitched back together to create the full rendering of the original sample. The physical sections then are available for further analysis; here we introduce the use of IMC to provide proteomic annotation of the tissue cells.

The challenge that has been overcome here is the creation of a robust and efficient analysis pipeline to take the digitized information from each imaging modality, and successfully integrating and aligning the various modalities. The registration is derived from techniques developed in astronomy, where a ground truth reference frame is defined by the marker beads embedded in the sample block. The visibility of the beads in all image modalities enables their use in several areas. The bead signature for each image section allows for the sorting of physical slices in silico and removes the requirement for the manual sorting of the STPT slice taken from the collection bath. By having the same beads visible in overlapping data segments, allows for effective stitching of large image mosaics. The STPT images provide a ground truth image reference frame into which all other imaging modalities can be reprojected.

The IMAXT processing infrastructure is able to integrate data from a range of image sources. The IMAXT Data Model is constructed to allow a full representation of the data to be captured in the associated metadata. Tracking of all processing steps is ensured through processing history updates to this metadata. The complete data processing software analysis infrastructure has been deployed on the IMAXT cloud, centralized on underlying hardware at the IMAXT Data Processing Centre in Cambridge. This cloud-based model enables the IMAXT collaborators to interact with the processed data products through a sophisticated science platform. The basic analysis pipelines generating the instrumentally calibrated science data products are essentially fully automated and able to process the large data sets generated by the IMAXT instrumentation suite. The federated data catalogues are provided as flat files, and also through a relational database. Subsets of the full data set are seamlessly streamed to the IMAXT Virtual Reality suite of tools^(^^13^^)^ for rich immersive visualization.

Future developments of the IMAXT analysis system will focus on the integration of additional modality-specific processing pipelines to allow the integration of additional transcriptomic and proteomic measurements, for instance from MERFISH and HiFi. Currently, the dissociated single-cell analysis informs the definition of the gene and protein panels used in the spatial imaging modalities. In the near future, the integrated 3D annotated tumor models will also allow feedback to single-cell studies, for instance, relating spatial clonal evolution in time-sampled Patient-derived xenograft (PDX) models with tracking of allele- and haplotype-specific copy number aberrations at single-cell resolution.

This will lead to richer annotated tissue samples at the sub-cell level and provide the underpinning data for spatial “omics” studies where the understanding of not only the biological make up at the cell level, but also the spatial context is required. The IMAXT analysis infrastructure represents a significant step forward in underpinning experimental imaging advances, and coupled with IMAXT’s novel VR-enhanced visualization and data immersion tools, will lead to paradigm shifts in our understanding of tumors and their environments.

In summary, we present the IMAXT data architecture including detailed descriptions of the analysis chains developed to enable the construction of accurate (to subcellular precisions), molecularly annotated, breast cancer models constructed from high spatial resolution STPT and IMC imaging.

## 4. Methods

### 4.1. Data analysis infrastructure

The requirements of a data analysis infrastructure able to process and analyze the vast amounts and variety of data for this project are: (a) being able to run noninteractive data pipelines specific to each modality in an automatic fashion, that is, as soon as data become available; (b) running user analysis batch jobs with specific resources allowing for resource scaling; (c) being able to perform interactive analysis that runs close to where the data are, and (d) running highly parallel optimized workflows. Together with these we also need in place on demand user storage, as well as data access processes and policies. The architectural approach taken has heritage in systems developed to handle optical and near-infrared imaging and spectroscopic data in astronomy, for example, VISTA^(^^14^^)^ and WEAVE^(^^15^^)^.

#### 4.1.1. IMAXT Cloud

The aim of the IMAXT Cloud is to allow users to run data analysis pipelines remotely and to work and analyze data interactively close to where the data are located, using already available software packages and utilizing computer resources as required. The core of our analysis platform is a Kubernetes (https://kubernetes.io) cluster that runs on premises. This cloud architecture allows for the flexibility required to allocate resources and scale jobs as needed. It allows us to treat all physical computers of the cluster as one unit as well as trivially scale up new hardware, deploy isolated applications, provide dynamic resource provisioning, and crucially maintain as good degree of stability and availability.

The IMAXT Cloud is deployed in our own dedicated hardware. However, it is worth noting that we use similar approaches to commercial Clouds. The system could be deployed with a few configuration changes in the Google Computing Platform, Amazon WebServices, Microsoft Azure, or any cloud that provides access to a Kubernetes cluster.

#### 4.1.2. Interactive analysis

The main language used for data pipelines, analysis, and infrastructure (archive, notebooks, background scripts, web services) across the project is Python^(^^16^^)^. Python has been acquiring great popularity across all modalities of industry and is indeed one of the main programming languages used in data science. Additionally, R^(^^17^^)^ and RStudio^(^^18^^)^ are offered for interactive analysis and user batch jobs. Interactive analysis is powered by Jupyter Notebooks^(^^19^^)^ which are spawned in our cloud on demand using JupyterHub (https://jupyter.org/hub). By navigating to a website the user will be taken to a live Jupyter notebook session from where they can run interactive analyses and visualizations. For ease of use, the environments have a large selection of the most widely used packages already installed.

It is worth noting that software environments are the same for all users, which improves shareability and reproducibility of analysis.

#### 4.1.3. Remote desktop environments

In order to facilitate different types of data access and analysis, we also provide access to on demand remote desktop environments. This allows the user to use the cluster as a remote machine with a user-specified request of resources, and access to preinstalled analysis and visualization tools (Ilastik, Cellprofiler, QuPath, Fiji, etc.).

#### 4.1.4. Batch jobs

Batch jobs are powered by a custom job scheduler that powers automatic data pipelines and allows users to submit their own analysis to the cluster. Data analysis jobs can be submitted using a command line from anywhere (i.e., users do not need to connect to a login node) and require no experience or technical skills.

#### 4.1.5. Parallel and distributed computing

We use Dask^(^^20^^)^ for parallel and distributed jobs. This allows for worker nodes that are provisioned on demand and can scale up or down depending on the workload needs. Dask also allows us to distribute large datasets across many nodes and perform efficient computations in parallel. Coupled with efficient data formats that can read and write chunks of data in parallel and independently allows for highly efficient analysis tasks to be performed on larger than memory datasets. These analysis tasks can automatically scale from a single computer to a 100-node cluster.

#### 4.1.6. Data model and data formats

The IMAXT project involves a large number of different data formats used across different modalities, some of which are created ad hoc for some instruments. Among the most common data formats, we find TIFF (Tagged Image File Format), HDF5 (Hierarchical Data Format), and CZI (Carl Zeiss Imaging format). This file format fragmentation is a real issue, as demonstrated by the fact that the Bio-Formats^(^^21^^)^ software tools incorporate plugins to read more than 150 proprietary file formats.

As for any large-scale multi-modality project, it is necessary to standardize the data model and format(s) to control and streamline data handling, bookkeeping, and to make it accessible to all users.

The other component of the data model is the metadata. We define metadata as any information relating to the biological image/data that is potentially required for its scientific analysis. That is not only image-related data like the dimensions and pixel scale of the image, but also the information on the instrument/microscope, the biological sample, and preparation/processing of the sample. Storing all metadata together with the data is necessary for proper bookkeeping and preventing any related human error. The metadata from different IMAXT modalities are included in separate files and/or not stored in a standard format. More importantly, the information on the sample or related preprocessing is often missing and only stored by the lab users in different formats and media.

Early in the development of our infrastructure, we identified that we needed a data format that allows for parallel reading and writing of different chunks of a dataset. This data format should also be able to contain both image data and metadata, to avoid any user misidentifications as data moves from one node to the other. It needed also to be flexible on metadata it can hold, as we have different modalities and instruments with a different range of metadata. These reasons lead us to choose a new emerging data format called Zarr (https://zarr.readthedocs.io). In Zarr datasets, the arrays are divided into chunks and compressed. These individual chunks can be stored as files on a filesystem, as objects in a cloud storage bucket or even in a database, making it efficient for clusters of CPUs to access the data in parallel. The metadata are stored in lightweight .json files and allows all the metadata to be in a single location which requires just one read.

All input data are then converted to Zarr, and all the metadata available stored within the dataset. All our pipelines work exclusively on Zarr datasets. We also welcome more recent developments where Zarr is the base specification for storing bioimaging data in the cloud^(^^22^^)^. We do as well provide custom converters from Zarr to pyramidal OME-TIFF^(^^23^^)^ since this is a widely supported format of many external tools. Our data conversion tools are available as open-source Python packages.

Information about the available data, data products, and their metadata are ingested into a relational database that allows interrogation via the IMAXT Web Portal (https://imaxt.ast.cam.ac.uk). Any future user updates to the metadata are tracked using versioning.

#### 4.1.7. Image and processing parameters

The typical size of a final data cube at the original resolution is around 2 TB made from 100 slices each of them made stitching 100 tile images. The largest samples we have processed are about 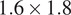 cm 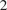 and up to 3 TB. Typical imaging parameters are summarized in Table 1. The time that it takes to run the full stitching and registration pipeline and produce the final cube varies depending on the number of CPU cores allocated for the process, and the speed of the disks. The choice of resources is given by the requirement to process a sample in about the same time it takes to acquire it. Using a typically 100 cores and 3 GB of RAM per core we get a processing time of 8 hr for a sample of 100 slices. Disk I/O performance is important since we compute a few intermediate files that we save to disk. Using solid-state disk storage reduces the overall time by a factor of two.

**Table 1.** Typical imaging parameters.

Instrument	No. of slices	Slice thickness (μm)	Pixel size (μm)	Sample size (cm)	Cell size (μm)
STPT	100–200	5–25	0.56		10–15
Axioscan	100–200		0.65–1.3	1–2	
IMC	10–100	15–25	1		
MERFISH	1–5		0.08–0.11	0.5	

*Note.* Typical physical slice thickness is 15 μm but STPT is able to acquire optical slices with a step of 5 μm.

#### 4.1.8. Automatic data analysis pipelines

As discussed above, many of the imaging data requires an extra level of processing to make them scientifically usable and to extract relevant information from them, from stitching to registration to segmentation. One of our main aims has been to build data pipelines that run in a fully automatic way, that is, once a dataset arrives to our storage it is processed without human intervention and without delay.

The IMAXT data flow diagram is shown in the Supplementary Information. Data taken with different microscopes are manually transferred to a specific storage location monitored by an uploader application. The uploader automatically transfers the data to the IMAXT cloud storage using Amazon simple storage protocol where they are converted to Zarr format. The metadata is also written into the IMAXT database with a versioning system that makes it possible for future updates. Once the conversion is done, the relevant data analysis pipeline is triggered automatically.

The results include metadata that allow their traceability, that is, among others, versions of the software and pipelines used, data provenance, parameters used in the processing, and so forth. All this information is ingested into the IMAXT database.

### 4.2. Stitching pipelines

STPT is a high-throughput 3D fluorescence imaging technique. It is similar to blockface imaging with the advantage of the images being relatively well aligned after acquisition, with measured slice-to-slice relative alignments below 20 μm. Note however that acquiring multiple slices introduces a drift in the microscope and the overall alignment between the first and last slice can be up to a few hundred microns. However, STPT has various advantages over standard blockface imaging. The tissue is imaged a few microns below the block surface, thereby limiting tissue deformations resulting from the cutting process^(^^1^^)^. Furthermore, by using a blade vibrating microtome instead of a milling machine, the sections can be used for further processing downstream using additional imaging modalities. The scanning operation is that of a regular two-photon microscope. A laser beam is used to excite one point in the sample, and the fluorescent photons are collected by the optics into a photomultiplier. The measured voltages are digitized and stored. Following the standard notation in astronomy, we will refer to one of these digital units as a count, measured in analog-to-digital units (ADU). Knowing the gain of the instrument allows to transform back from ADU to electrons, and from there given a quantum efficiency into incident photons^(^^24^^)^.

An image of the field of view of the microscope, hereafter a tile, is constructed by scanning with the laser across the sample and measuring the excitation intensity produced by the laser at each point, encoded by the microscope as a pixel, with a typical resolution of 0.56 μm per pixel and a size of 2,080 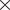 2,080 pixels. Different points in the focal plane are scanned by changing the angle of the incident beam. Because sample sizes are larger (typically, by a factor of a hundred) than the field of view of the microscope, we need to acquire several tiles in order to map the full staging area. Once a tile is acquired, the stage is moved in order to image consecutive tiles at the same optical depth. Tiles are acquired with an overlap of 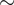 10% (this is a configurable parameter) to allow for stitching (see below). Once the whole sample has been scanned, the microtome cuts the top slice and the process is repeated at a deeper surface into the sample. For a typical sample used in IMAXT, 100 to 300 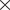 15 μm thick slices are acquired in this fashion.

These sectioned slices fall into a bath and are collected manually and deposited onto a glass slide once the sectioning/imaging process is complete. Because this effectively randomizes the order of acquisition, each slide is then imaged using an Axioscan and labeled. Federating each of these Axioscan images with the STPT image cube ensures traceability and the ability to reconstruct 3D volumes from the data from other modalities. This Axioscan microscope uses a CMOS detector to acquire fluorescence imaging of the slice (again using a tiling pattern to map the full slice) in a range of channels at different wavelengths. The Axioscan however images the top surface of the sample as it is deposited on the glass slide. This introduces some degree of complication in the Axioscan to STPT matching, as the latter images are of a thin optical layer some microns deep into the sample, and furthermore, when depositing the slice onto the glass slide this will be randomly done “bottom-up” or “face-up.”

**Figure 5.**
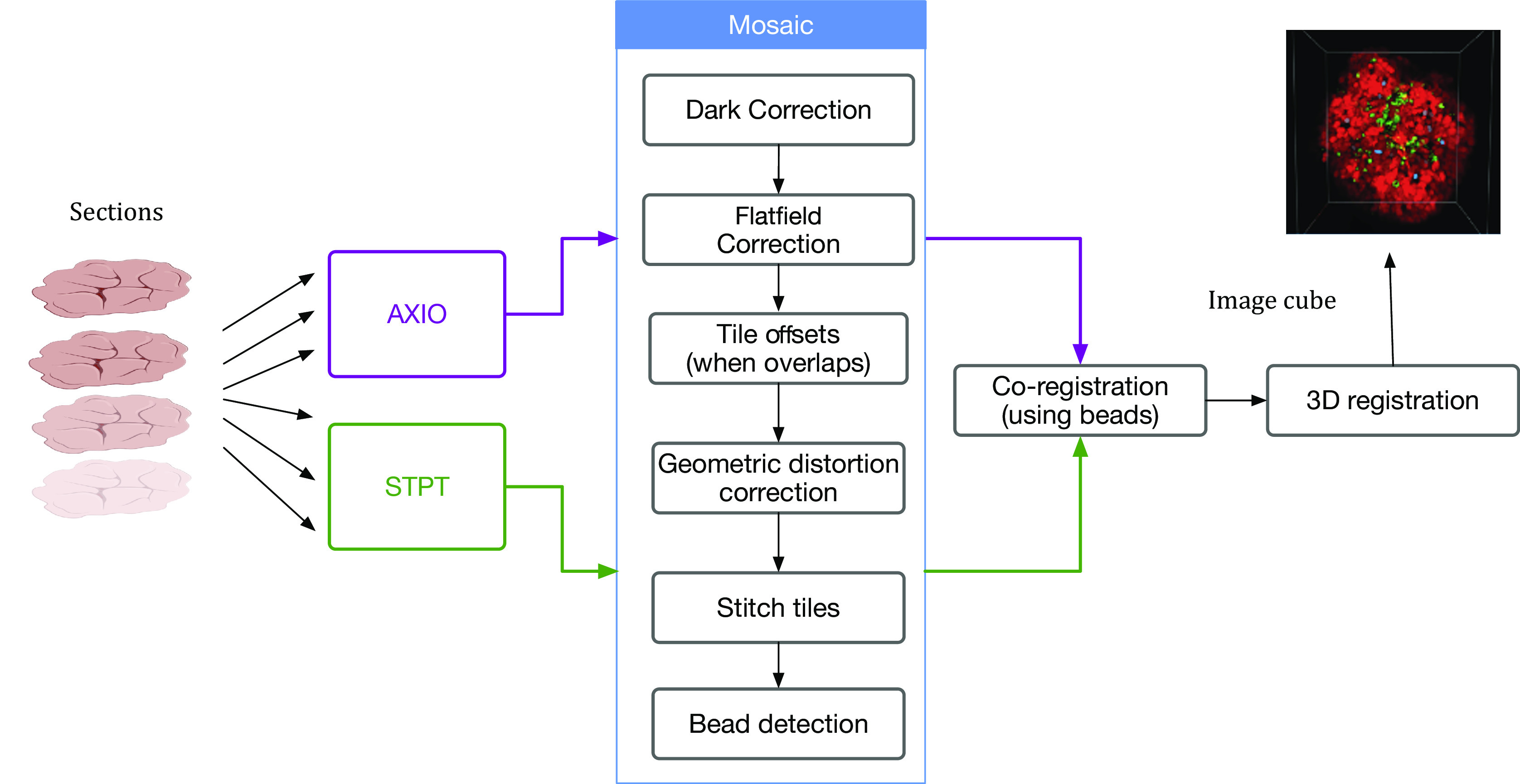
Processing pipeline steps applied to both STPT and Axioscan raw image data. Note that in the experimental flow the STPT acquires the images of the slides and does the sectioning after which they are acquired with Axioscan.

Despite the differences between both modalities, from a data processing point of view the software needs are quite similar and thus we bundle them together in this section. In order to produce science-ready images, we need to stitch all the single tiles into a mosaic that encompasses the whole stage. Before doing this, though, we will try to remove the instrumental effects present in the images, namely: dark current, flat-field correction, and optical distortion. Figure 5 shows the steps involved in the image data stitching pipeline.

#### 4.2.1. Dark current correction

Strictly speaking, dark current is associated with thermal noise generated in the detector itself that is added to the recorded signal. Measuring dark current on-sample is difficult, and dark frames are usually generated by taking images under similar conditions (exposure time, temperature, etc.) but with no light reaching the detector. When these calibration frames are not accessible, statistical approaches allow for disentangling dark current and signal (e.g., BaSiC^(^^25^^)^).

**Figure 6.**
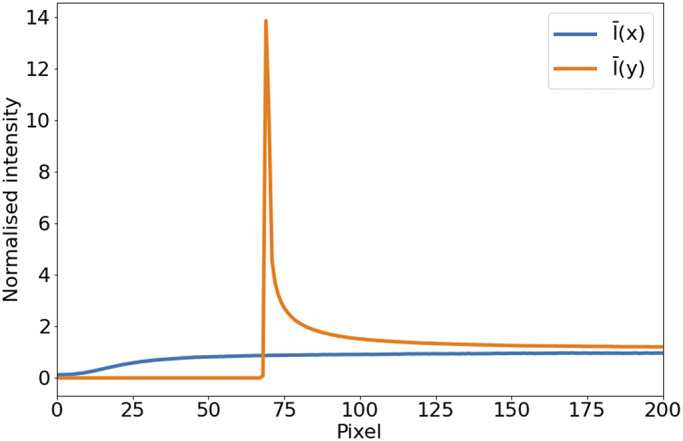
Average intensity per pixel. Normalized intensity averaged over columns ( 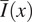 , blue) and rows ( 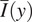 , orange) over the first tenth of the detector. The effect of background illumination is evident here. The first 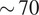 pixels are already truncated by the microscope software to eliminate the most contaminated areas.

In the case of STPT, since the detector is a single-pixel photomultiplier, thermal noise is just an additive constant to the images (we have found no evidence of thermal drift within a single tile). Background illumination and stray light are a bigger source of contamination and can be seen in median-stacked frames (Figure 6). This contamination is only relevant in the borders of each tile, and since we always use overlapping tiles, we can get rid of pixels with high background without loss of information. For the Axioscan, background and thermal signals are very low. We, therefore, do not apply dark current subtraction to these modalities, although our pipeline has the capability of measuring and correcting these additive terms.

#### 4.2.2. Flatfield correction

In the case of most 2D detectors, the quantum efficiency is not constant across all pixels, and inhomogeneities in the lenses and other factors (like dust particles) result in a transmissivity that is a function of position in the field of view of the instrument. The standard way to measure these effects is to take the image of an uniform light source. By normalizing this image, we measure the per-pixel response function for the system at a given wavelength. Our experimental design leads to 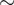 100 tiles per slice, and several tens of slices per sample. If we stack all these tiles, each pixel sees a random intensity distribution that, given enough tiles, should be homogeneous over the field of view. Therefore, any variation of a location statistic like the median or the mean across the field is a measure of the different response of the system at each pixel.

Applying this correction to Axioscan tiles is straight-forward. STPT uses an unidimensional detector, so there are no pixel-to-pixel differences in response, but as the laser systematically illuminates different parts of the field of view, if effectively maps different light-paths along the optical system that can have different throughput, leading to the need to intensity correct each tile to homogenize the overall response.

**Figure 7.**
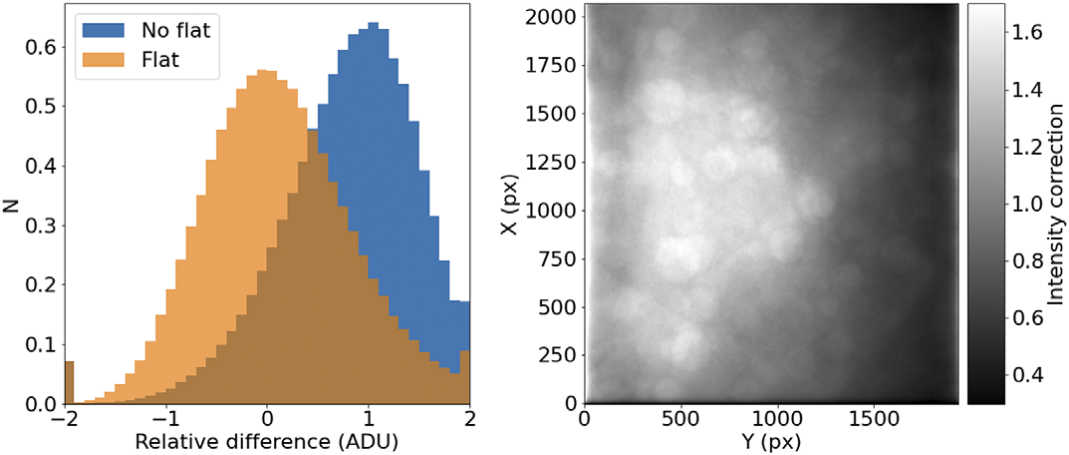
STPT flatfield correction. *Left*: Relative difference of overlapping pixels before (blue) and after (orange) flatfield correction. *Right*: Example of normalized flatfield frame.

It should be noted that, for a given wavelength and objective, this correction should remain relatively constant over times of days or weeks. This allows us to use this procedure with samples that may not reach enough tiles to offer reliable statistics (Figure 7).

#### 4.2.3. Distortion correction

**Figure 8.**
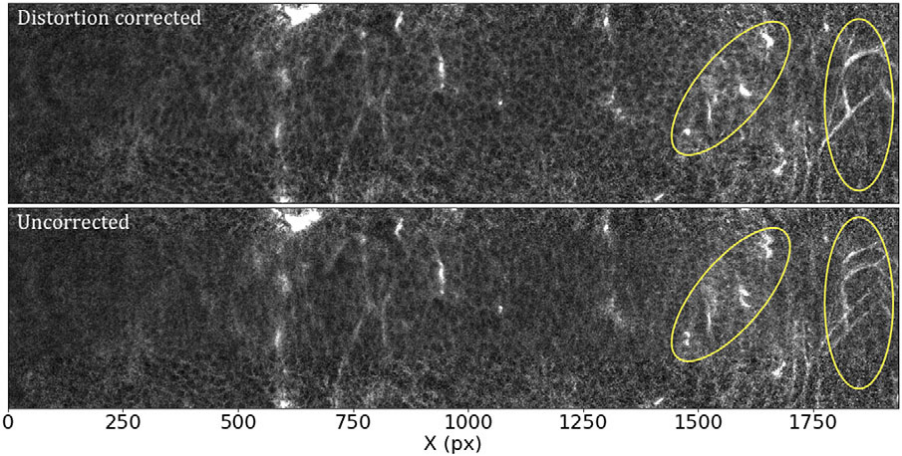
Effects of the distortion correction as applied to STPT tiles. Both panels show the same patch (500 px across in the *Y* direction) of two overlapping tiles. The ellipses highlight areas where distortion effects are most visible. *Top:* Distortion-corrected overlap. *Bottom:* Uncorrected overlap.

Most optical systems are subject to optical distortions. These manifest as a change of scale (resulting in a change in the shape of cells) in the field of view, being normally negligible in the center and increasing with the distance to the center of the field of view. This effect is particularly notorious when comparing overlapping tiles, as can be seen in Figure 8: while toward the center of the field of view the undistorted tiles align well, as we move toward the corners, features start to blur, to the point that they appear to duplicate close to the corners of the array.

**Figure 9.**
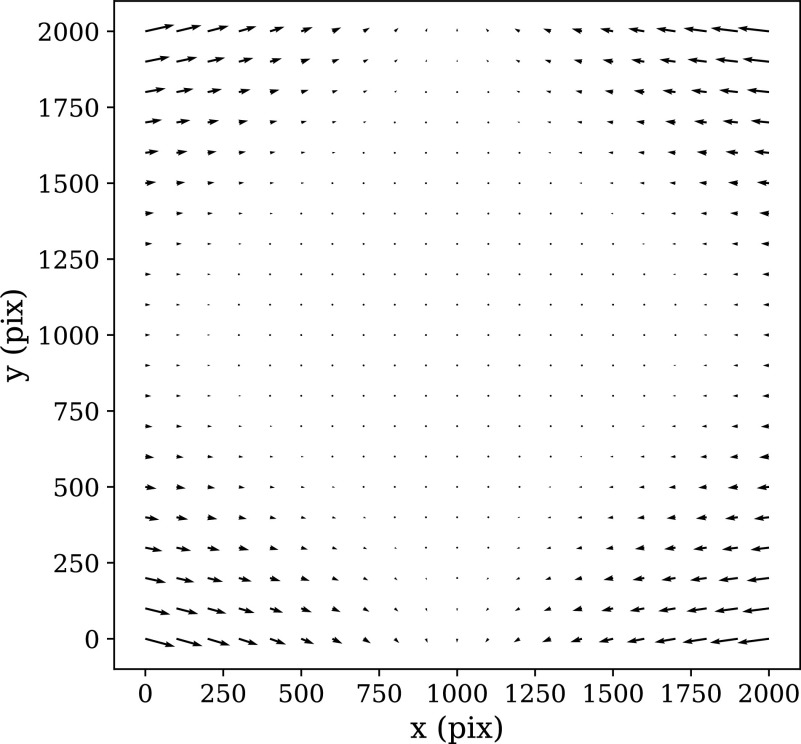
STPT geometric distortion correction. Field of view optical distortion for STPT produced by the optics of the microscope. Maximum distortion in the corners of the image is of the order of 20 μm. This is corrected before the registration between tiles by resampling the images to an undistorted pixel space.

In order to calculate the optical distortion of the STPT optical system, we acquired a calibration sample consisting of 10 slices in which the overlap between tiles was 50% of the field of view both in X and Y. In this configuration, each point of the sample is mapped by at least two pixels, and using feature-rich parts we can minimize intensity differences between these pixels in order to fit the optical distortion of the system. The best model is a polynomial of grade 3 with different magnifications in *X* and *Y.* The resulting distortion (see Figure 9) is a stable characteristic of the instrumental setup and used to correct the tiles for all scans of the same instrument, although it needs to be recalibrated for each focal lens.

In the case of our Axioscan, the optical distortion is small and is left uncorrected.

One issue worth noting is that the microscope produces square tiles, and after processing, the pipeline uses rectangular tiles. Because applying the distortion map in Figure 9 effectively “squeezes” the pixels close to the corners of each tile, distortion-corrected tiles have some empty pixels. In order to keep account of this, we use a construct common in astronomy called a confidence map. This is just a weight image with the same size of a tile that encodes the provenance of each pixel. In this case, this will be a value of 0.0 for these empty pixels and a value of 1.0 for all the other ones.

#### 4.2.4. Registration between tiles

Because the STPT microscope sees the biological samples before slicing and after minimal manipulation, imagery coming from this modality constitutes the stepping stone of much of our analysis. It is crucial that the science-grade images produced by our pipeline are the best possible and most precise representation of the sample. The control software of the microscope records the absolute position of each tile within the stage and uses this to reconstruct a raw full-stage image, but we have found that there are small positioning errors that lead to a poor reconstruction of the full image. This, coupled with the fact that the microscope software does not correct for flatfield or optical distortion, merits an improvement of the full-stage reconstruction from individual tiles. We refer to this process as stitching.

As we have discussed before, in our configuration, STPT tiles always overlap by 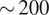 px. Starting with the recorded position provided by the microscope, we can find which tiles are neighboring each other. Once tiles have been distorted and flatfielded, we can use overlapping pixels and find the displacement that minimizes intensity differences, using as weights the confidence maps generated in the previous step. In order not to do subpixel resampling at this stage we also assume that the displacements are integer pixel values. This method achieves similar results as the commonly used phase correlation methods using Fourier transform^(^^26^^)^ but correctly taking into account masked arrays^(^^27^^)^. Typical differences between displacements coming from the microscope and the ones calculated on-sample are of 5–10 μm for STPT, and of around 1 μm for Axioscan. Because these differences are of the order of the expected crossmatching error, in the case of the Axioscan we use the displacements provided by the microscope.

Once we have the pairwise offsets between adjacent tiles, we need to compute the absolute position of the tiles in the full stage. We do this by using as reference the tile with the highest average intensity. This tile is always one that sits within the biological sample. Using the relative displacements we lay its four neighboring tiles, and compute their absolute position. We now use each of these as reference and repeat the process iteratively until all tiles have a calculated absolute position. Because in this way there is more than one tile-laying sequence for most tiles, we average the absolute positions weighted with the positioning error.

It should be noted that this method (as any other intensity-based registration) only works where there is enough information in the overlapping regions. For pairs of tiles that have empty overlaps (either because there are no visible beads or sample tissue in this region), we retain the displacements coming from the microscope. This is directly related with bead density in the sample substrate. For low-density samples, a high fraction of tiles will have empty overlaps. By starting the stitching from the sample outwards, we reduce this effect: all the tile overlaps in the sample will have enough information for a good matching, and this extends to beads that are up to 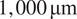 from the sample (as an STPT tile is roughly 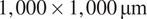 ).

**Figure 10.**
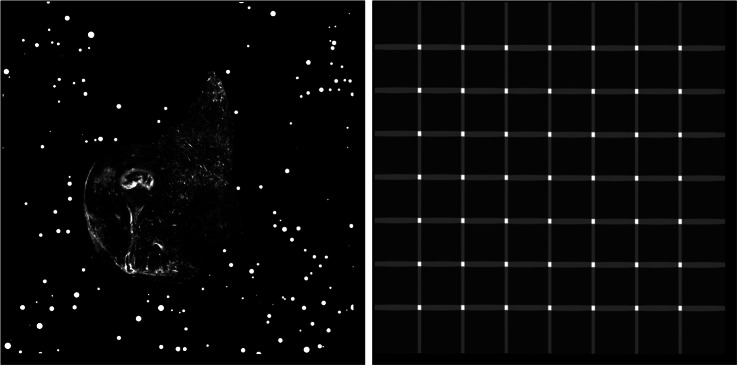
Example final stitched STPT stage mosaic (left) with associated confidence map (right). Images are padded so that all slices from a sample have the same size, hence explaining the pixels with zero confidence at the right and lower ends of the confidence map.

With these new absolute positions, we calculate the stage size, in pixels, and fill this image by adding intensity values from each pixel of the individual tiles. We also generate a full-stage confidence map by projecting individual confidence maps in a similar manner. Dividing the intensity image by the confidence map takes care of averaging overlapping regions, and the final science ready image is stored (Figure 10). Note that thanks to the flatfield correction, there is no need of additional nonlinear intensity blending in order to remove tile border effects.

#### 4.2.5. Registration between slices

**Figure 11.**
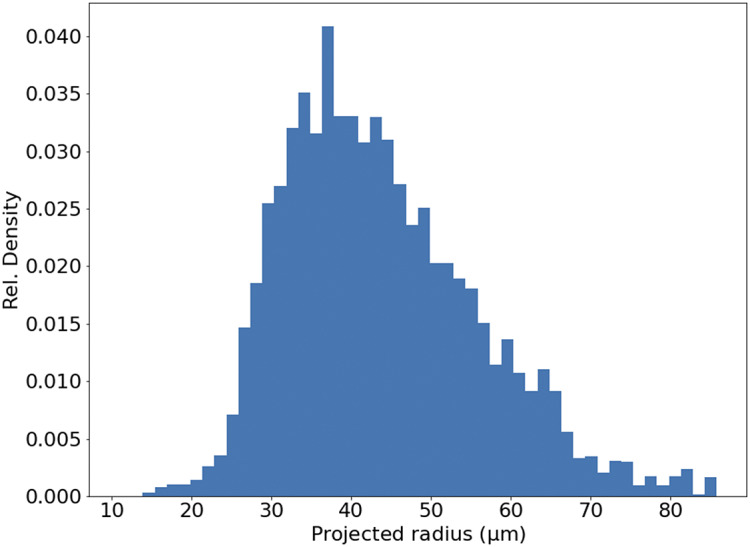
Histogram of projected radius of a sample of randomly selected beads. The projected radius is the apparent radius of a bead in an slice image.

In theory, the serial sections produced by STPT are inherently aligned. In reality, for each microtome pass the entire stage may shift, and some misalignment may be introduced. This can be also be due to the fact that we stitch each slice independently. Because we need to reconstruct the 3D sample as seen by the STPT microscope, we need to make sure that the relative alignment of the slices is consistent. Naively, one could think that intensity-matching slices could solve this problem, but because each microtome cut removes 15 μm of sample and the STPT focuses a few microns below the sample surface, this is not possible, as images are far apart in sample depth. In order to solve this (and allow for registration across modalities further down the data processing), we introduce spherical beads in the sample. These beads have a typical diameter of 90 μm (Figure 11) and therefore can be clearly seen in several consecutive slices. While the outline of the beads changes between slices, their center remains constant with depth (within the natural experimental limitations of sample manipulation, microtome blade sharpness effects, etc.) and can be used as fiducial marks. This effectively transforms the circular cutouts of the spherical beads into point sources, and opens the problem to the application of a wide library of algorithms inherited from astronomy, as locating and crossmatching the position of point sources is a problem underlying many astronomical applications.

**Figure 12.**
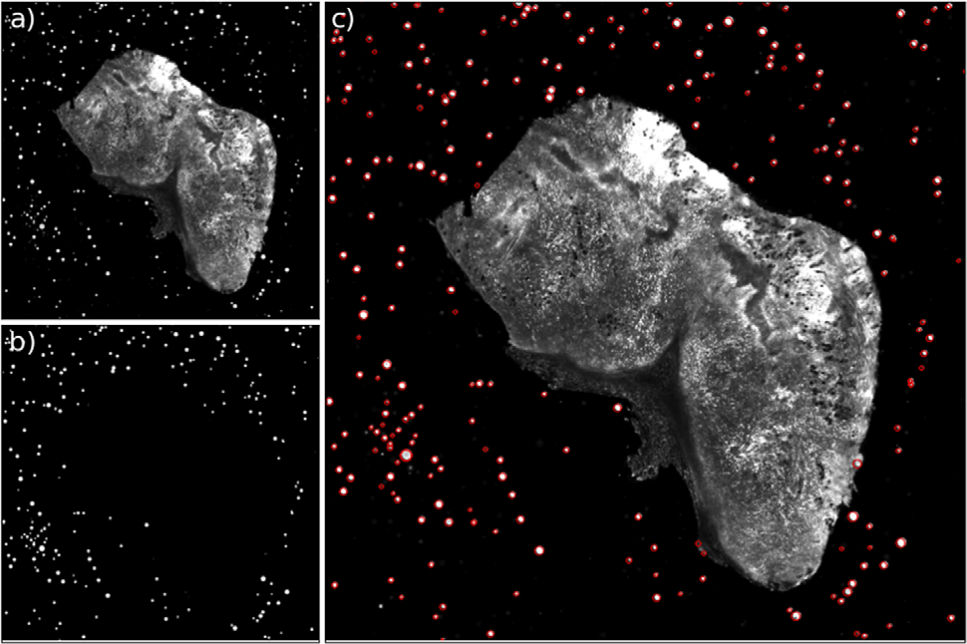
Example of the bead detection and profiling for an STPT mosaic. Panel (a) contains the original STPT image, (b) depicts the bead detection mask produced by the U-Net network, and (c) depicts the original image plus the fitted radius for each detected bead.

The first step in our registration algorithm is to segment the beads. We use for this a U-Net neural network^(^^28^^)^, trained over a set of manually segmented images.^1^ This network produces a bead detection mask, and we use watershed segmentation to differentiate between individual beads, and produce a label mask (Figure 12). This label mask still contains a small number of false positives, in particular for small fragments of sample. These will be filtered out downstream when fitting the bead profile, as they offer poor fits to functions with polar symmetry, and often end with fitted radii that are too large for a realistic bead.

We have processed around 200 samples in total each with 100 to 200 slices. The number of false positives beads in each slice is about 1–3% (Table 2). False positives give unrealistic fitted radius (i.e., larger than 5 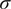 the average).

**Table 2.** Statistics on number of beads for some random STPT slices.

Slice no.	No. of beads	Average radius (μm)	Dispersion (μm)	False positives (%)
1	332	47	11	0.90
2	327	49	11	0.61
3	401	46	10	1.75
4	327	48	12	0.31
5	406	47	10	1.48
6	348	45	11	1.15
7	360	46	10	1.67
8	347	46	10	1.73
9	360	46	10	2.78
10	354	46	9	3.67
11	330	47	10	1.52
12	333	44	10	1.20
13	352	46	11	0.57
14	377	46	10	2.12

*Note.* For each slice, we show the total number of beads detected, their average radius, the dispersion of the radius values (standard deviation) and the percentage of false positives defined as the beads with radius larger than 5 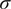 the average.

Although there are simpler ways to separate isolated spherical beads from a large, continuous biological sample, our U-Net based algorithm has several advantages. Firstly, it is a relatively simple architecture that does not add much overhead in the way of code compared to other mathematically simpler segmentation algorithms. Secondly, to extend the pipeline to a new modality, we just need to train a new network (in case beads appear significantly different) without having to alter any code at all. Thirdly, for fragmented samples or those with complex tissue distributions that lead to spotty images, the network offers better segmentation than contrast-based algorithms. This is also true for beads that are almost adjacent to the sample. It should also be noted that this segmentation does not require any manual intervention or supervision, making it ideal for a fully automated pipeline like ours.

In order to derive the coordinates for the bead centers, we build a simple but physically realistic model of the beads consisting of:

- A sphere of constant emissivity and radius 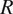 centered in coordinates 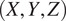 as measured with respect to the 3D sample block.
- The STPT laser excites a layer of thickness 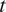 of the bead at a given depth 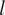 into the sample, so that the emission from this layer in local coordinates 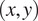 is 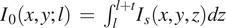 . In this case, as we assume emissivity is constant over the sphere, 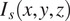 is just the density profile of a sphere with constant density in Cartesian coordinates.
- The depth 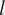 can either be in the interval 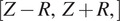 and so the optical surface intersects the bead, or 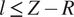 in which case we have a fully embedded bead emitting just under the optical surface. If 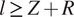 , the bead is between the optical surface and the observer and therefore not visible.
- The sample substrate has an optical depth 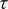 , and the emission from the optical surface decays as 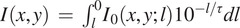 .
- By integrating this last expression we obtain the 2D brightness profile as a function of 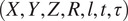 that we fit to the observed profile assuming Poisson statistics.

**Figure 13.**
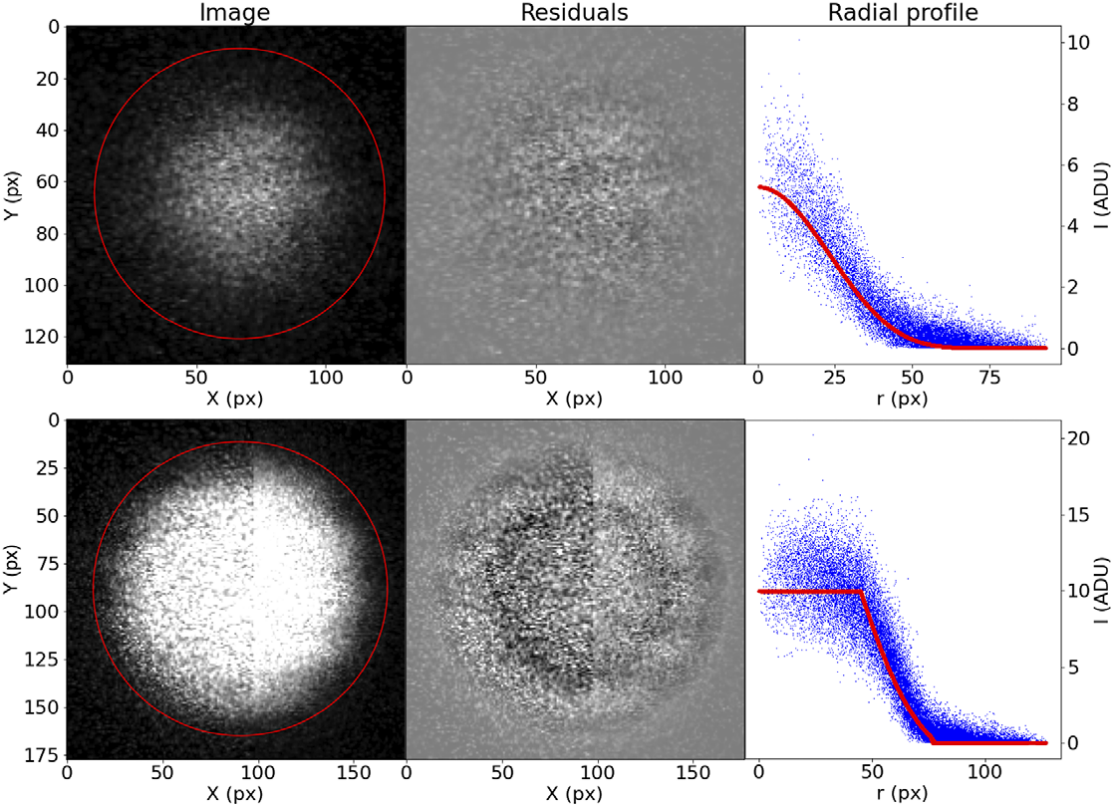
Examples of the profiling function applied to measured STPT beads.

Although this prescription may seem too complicated, it accommodates well the variety of bead brightness profiles observed in our samples (Figure 13). For the purposes of registration, the most relevant parameters are 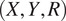 . The coordinates of the bead center are the basis of our registration, and the bead radius is a simple threshold to use when finding the best matching beads between slices.

Once we have the 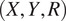 catalogue for all beads and all slices, we can proceed to the slice-to-slice registration. Because inter-slice displacements are expected to be small, for each pair of consecutive slices we find the best bead matches by using a simple nearest-neighbor search. With all the matched beads, we identify unique beads. Normally, due to their thickness, each bead will appear in a handful of slices. For a bead detection to be considered, we require that it appears in at least two physical slices (and the associated optical slices). This filtering of single detections removes the vast majority of spurious contaminants left, as in can be seen in the third panel of Figure 12.

With all the detections of a single bead 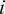 over all slices, we can compute the matrix with all the pairwise differences in coordinates, 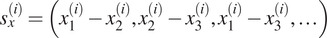 , with 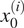 being the fitted 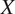 coordinate for the center of bead 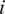 in the first slice, and so on. Accumulating all beads and all slices, we build the vector 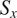 . We can relate this sample vector with the vector containing the absolute offsets for the slices 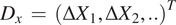 by means of a coefficient matrix 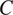 :

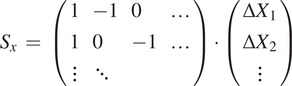




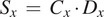

And

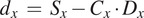

**Figure 14.**
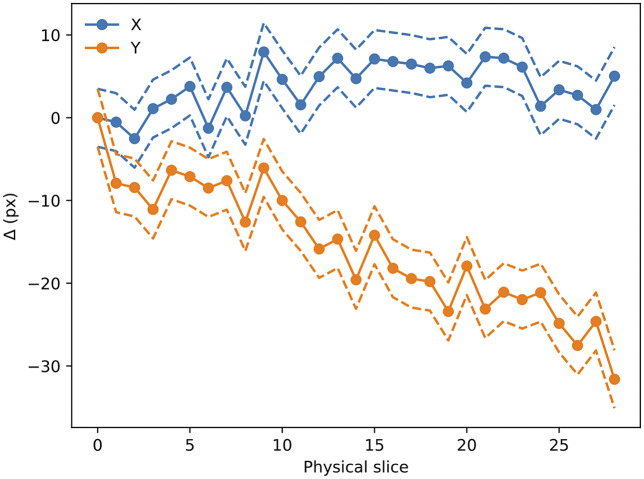
Evolution of 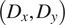 with slice number (i.e., depth along the sample). Dashed lines mark the 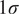 error boundary. As can be seen, there is a drift in one of the directions, likely to be related to the effect of the microtome blade pushing into the sample cube. The scale for this sample is 0.56 μm per pixel.

We can obtain the absolute displacements 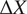 by minimizing the equation

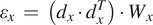

with 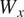 being a set of weights derived from the error vector for the pairwise differences in 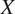 coordinate for the centers. Although 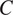 can be a relatively large matrix, it is sparse, and therefore this minimization is computationally efficient. The same scheme is applied to 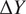 (we assume displacements in both axes are independent) and from 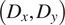 we can obtain the full 3D registration of the STPT cube. An example of derived 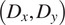 can be seen in Figure 14; while 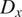 remains more or less constant, a clear drift can be observed for 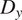 . This is likely related to the effect of the microtome that always sections the sample along the same direction, possibly causing small displacements.

The 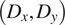 translation values are stored in the metadata of the Zarr file and are read when processing the STPT images.

**Figure 15.**
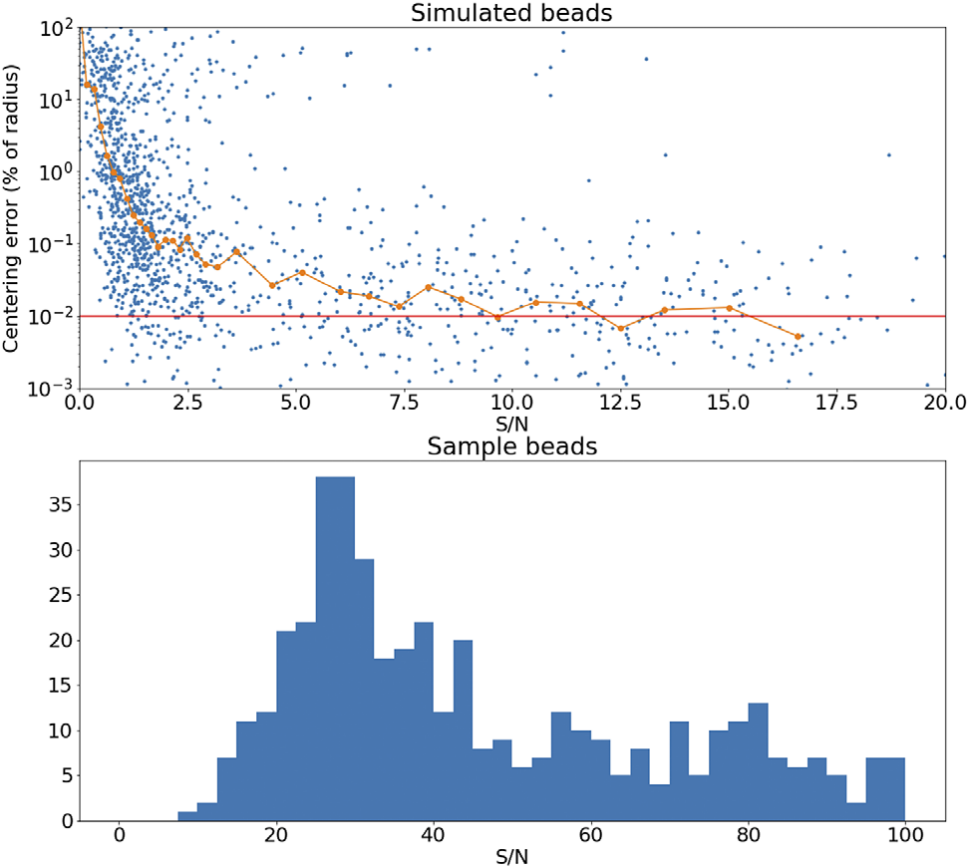
Top panel: Error in the recovered center coordinates for beads simulated at different S/N, as a fraction of the bead radius. The orange line represents a running median. At 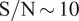 the median error reaches the asymptotic value denoted by the horizontal red line. Bottom panel: histogram of the measured S/N for a random sample of beads.

**Figure 16.**
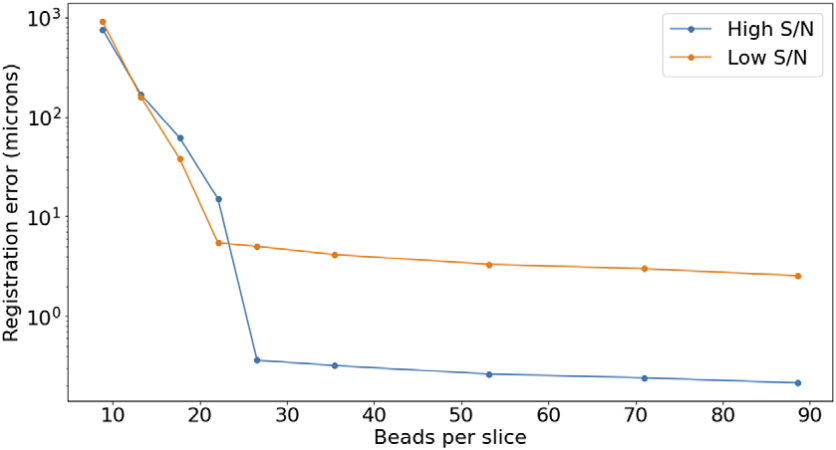
Registration error as a function of the average number of beads on each slice. The high S/N regime corresponds to 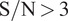 , while low S/N stands for 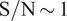 .

The accuracy of this registration depends strongly on the number of available beads. The modalities discussed here have pixel scales between 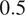 and 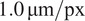 , and so the typical bead will be sampled over many pixels. It follows that the detection and profiling are robust with signal to noise: centering errors below 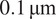 can be achieved down to S/N 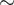 2 (Figure 15), but the quality of registration decays strongly for low bead density. While this slice-to-slice registration only fits for two free parameters, more generally (as will be discussed later) 6 free parameters are needed. For this more complex model, at least 20 beads per slice are required (equivalent to about 
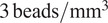
 ) in order to achieve precision below 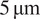 , as can be seen in Figure 16.

### 4.3. Axioscan and MERFISH cell segmentation pipeline

In recent years convolutional neural networks (CNNs) have become a popular method to segment cells. A common problem here is the separation of touching cells. Cellpose^(^^29^^)^ provides a solution to this problem by generating and predict a gradient in the horizontal and vertical direction for each cell. This results in clear borders between cells. Another way to distinguish separate objects is the use of nonmaximum suppression (NMS) of bounding boxes to select those regions with a higher confidence when they overlap. In the case of cells, a box shape would not suffice. Therefore, StarDist^(^^30^^)^ was proposed which instead uses star-convex polygons.

Both Cellpose and StarDist come with pretrained models for fluorescent images. However, these models do not generalize well to the Axio and MERFISH imaging data. We, therefore, opted to generate a new dataset of the imaging modalities used in the IMAXT project, in order to train new Cellpose and StarDist models. Images were acquired from 18 tissue samples of various healthy organs and tumor types of mouse models. From 13 of these samples, images had been acquired using Axio, while the remaining 5 samples had been imaged using MERFISH. Representative image regions were manually selected to generate a dataset that captures the wide range of variation in appearance of various tissue and cell types, including the particular challenging cases. The MERFISH images were furthermore down-sampled to accommodate the receptive field of StarDist.

For the manual segmentation of the cell nuclei, we recruited 8 volunteers. Each of these annotators was subsequently assigned one image from each of the 18 tissue samples, which have on average 305 cells per image. The annotations were performed using QuPath v0.3.x. Using this software, the annotator was able to have the annotations of the cells overlap. These were converted to images with nonoverlapping cells by generating a distance map for each cell mask and assigning pixels to a cell only when they have a larger value in the distance map compared to the other cells. The resulting manually annotated dataset of 144 images was split randomly into 128 for training and 16 for testing, while making sure the test set includes all modalities and tissue types.

For the training of the model, we additionally added publicly available datasets of fluorescent images with annotated cell nuclei to further enhance the generalizability of the model. These public datasets include BBBC020, BBBC038v1, and BBBC039v1 from the Broad Bioimage Benchmark Collection^(^^31^^–^^33^^)^. For BBBC038v1 we used the fluorescent images of “stage1 train” only, from the unofficial fixes by Konstantin Lopuhin (https://github.com/lopuhin/kaggle-dsbowl-2018-dataset-fixes). We also used the images from Coelho *et al.*^(^^34^^)^, which consists of hand-segmented nuclear images of 3 T3 and U20S cells.

#### 4.3.1. Performance

To evaluate the segmentation accuracy we calculate a pixel-wise and object-wise F1 score as previously proposed ^(^^33^^,^^35^^)^. Here, we consider a predicted and ground truth cell to match when their Intersection over Union is greater than 0.5. The mean 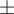 standard deviation of these measures are presented for our newly trained models (IMAXT Cellpose and IMAXT StarDist), as well as for the pretrained Cellpose model and the two pretrained StarDist models, in Table 3. These results indicate a clear performance increase of the newly trained Cellpose and StarDist models, compared to the pretrained models, with have a similar accuracy between them.

**Table 3.** Quantitative evaluation of the cell nuclei segmentation accuracy of the pretrained Cellpose and two pretrained StarDist models, as well as the new model Cellpose and StarDist models trained on the IMAXT and public datasets.

Method	Model	Pixel-wise F1	Object-wise F1
Cellpose	Nuclei	0.68 ± 0.24	0.59 ± 0.28
	IMAXT Cellpose	0.86 ± 0.04	0.79 ± 0.12
StarDist	2D paper dsb2018	0.73 ± 0.17	0.51 ± 0.29
	2D versatile fluo	0.80 ± 0.06	0.59 ± 0.22
	IMAXT StarDist	0.85 ± 0.03	0.76 ± 0.08

### 4.4. IMC segmentation pipeline

IMC is an approach to high-multiplex imaging and single-cell protein analysis^(^^2^^)^. The method delivers unprecedented insight into the tissue microenvironment with single-cell resolution. It helps to deeply characterize the complex and diverse tissue microenvironment by gaining an unparalleled understanding of the spatial relationships between a multitude of cell types and the role of cell phenotypes in the context of disease. It also helps to uncover novel therapeutic targets through the discovery of new biomarkers. In IMC, tissues are stained with a panel of isotope-labeled antibodies. Stained sections are laser ablated at 200 Hz at subcellular resolution, and liberated isotopes are detected with a mass cytometer to yield images quantifying the abundance and location of the proteins of interest at 1-micron resolution simultaneously. The output of this process is a data cube consisting of several layers where each layer is associated with a protein. In addition, all layers are aligned. Thus image registration as part of a preprocessing step is not necessary. The name of each protein and the order of its corresponding image layer is stored in the data cube metadata. In addition, there are few extra image layers related to the instrument calibration.

#### 4.4.1. Method

There are several approaches to segment cells in microscopic images. These include unsupervised, supervised, or a hybrid method. An example of unsupervised method is watershed algorithm which can be run by setting a few initial parameters associated with the size and configuration of the cells to be segmented. Watershed algorithm is fast and can be robust if dealing with high signal to noise image of cells where cells are not closely packed. However, it is less accurate in segmenting low signal-to-noise cell images or segmenting patterns such as cell’s cytoplasm or membrane. Such pattern are presented as separate channels multiplexed imaging data such as IMC. An accurate segmentation of cell’s nuclear and cytoplasm/membrane channels is necessary to correctly perform the cell/tissue type identification. With this regards, supervised methods achieve a better segmentation results in segmenting cell’s nuclei and cytoplasm/membrane, specially where cells are in compact configurations, by using pixel-level annotated masks to train a segmentation classifier. For instance, Ali *et al.*^(^^36^^)^ uses a hybrid workflow, that is, a combination of supervised and unsupervised methods to properly segment cells in IMC data. Their hybrid workflow uses Ilastik^(^^37^^)^ to create probability maps, from manually annotated data, and uses those maps as input to CellProfiler^(^^38^^)^ to perform water-shed segmentation of the probability maps. The creation of probability maps in Ilastik (supervised method) requires user inputs for model training. This is a time-consuming manual task which is usually done by experienced biologists or pathologists. This process becomes even more time-consuming when dealing with multiplexing imaging data such as IMC where the manual annotation needs to be done in more than one channel to capture the full extent of cells. In IMAXT, our aim is to create an automated end-to-end pipeline to analyze IMC data. Therefore to take full advantage of the hybrid approach, as discussed above, and to avoid performing manual annotation, we use the Deep Learning-Based Cell Segmentation for IMC DICE-XMBD^(^^39^^)^ CNN model to create probability maps. It is shown that a combination of probability maps produced by Dice-XMBD CNN model and watershed segmentation outperforms other methods for segmenting cells in the IMC data^(^^39^^)^.

#### 4.4.2. Creating probability maps

**Figure 17.**
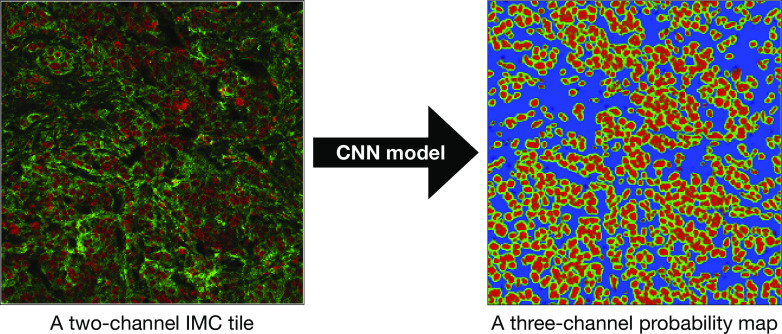
Dice-XMBD CNN model is used to convert a sample two-channel IMC tile (left) into a probability map (right). In both panels, red color is associated with nuclear marker and green represents the cytoplasm. The size of each tile is 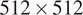 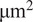 .

The first stage to segment IMC imaging data in the IMAXT IMC pipeline is to create probability maps using Dice-XMBD trained model. The input to this model is a 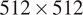 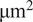 (IMC pixel resolution is 1.0 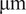 per pixel) 2-channel IMC image data cube where the first channel represents nuclear marker and the second channel is either cytoplasmic or membrane channel. Therefore, as a preliminary step, and to prepare the input data for the CNN model, the IMC image is divided into a series of tiles, each having a size of 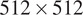 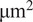 , with a stride of 50 pixels along 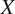 and 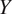 axes. In addition, during the preparation of IMC tiles, each channel is preprocessed and normalized. The reason is that the IMC data consists of 16-bit images enabling the instrument to map a high dynamic range of pixel values. However, the distribution of pixel values associated with the observed tissue mostly populates the low-intensity domain of the available dynamic range, that is, not taking full advantage of the dynamic range for a 16-bit depth image. Therefore, it is necessary to enhance IMC channels to be used with the CNN model. This is done by applying a linear scaling function to the image pixel values to improve their contrast by stretching the range of intensity values to span the desired values for a 16-bit image. This technique is called image normalization and is different from histogram equalization, where the scaling is nonlinear. For each input tile (2-channels), the CNN model outputs a 3-channels probability map (see Figure 17). Channels in the probability map are associated with background, cell nuclei, and cell cytoplasm.

#### 4.4.3. Segmenting probability maps

**Figure 18.**
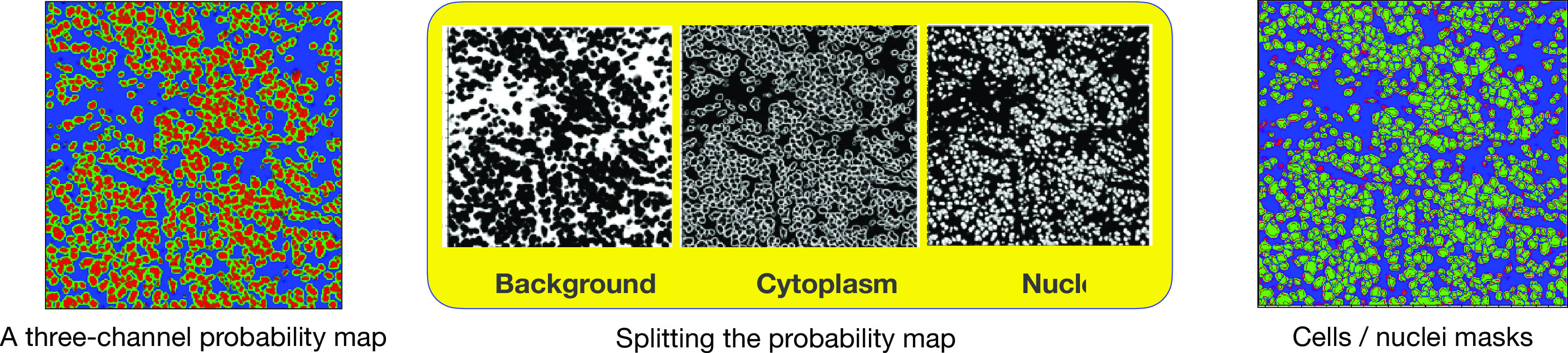
A probability map (left panel) is split into 3-channels associated with background, nuclear, and cytoplasmic channels (middle panel). The outcome of the segmentation of probability map is cells/nuclei masks (right panel). The size of each tile is 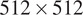 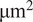 .

The second stage, in the IMC pipeline, is to segment modeled cells in the probability map. Since probability maps represent both cell’s nucleus and its boundary, they can be used to segment not only the nucleus but also the whole cell. To do so, a probability map is split into three channels, that is, background, nucleus, and cytoplasm. During the whole segmentation process of cells in the probability map, nuclear channel serves as a reference image. Thus, we initially segment nuclei in nuclear (reference) channel. First (a) a Gaussian filter, with an appropriate kernel size, is applied to remove individual high signal-to-noise pixels in the reference channel. Then (b) we separate nuclei from background pixels by applying a global thresholding method (e.g., Otsu). The output is a binary image where pixels belong to the regions of interest (nuclei) are white and consist of either individual cells or those having overlaps with one another. To deblend overlapping (touching) cells, (c) we find the coordinates of local peaks (maxima) in the binarized reference image by using distance transform algorithm. Then, (d) we use a watershed algorithm to segment the image^(^^40^^)^. Main outputs of this process are positions of nuclei in the probability map as well as nuclear masks (see Figure 18).

Next, to segment the whole cell, we repeat the same procedure (a) to (d) as discussed above but this time on a combination (addition) of nuclear and cytoplasmic channels. One very important difference however is that during watershed segmentation, that is, step (d), we feed nuclei positions as initial seeds to the watershed algorithm. This ensures that we find as many segmented cells as nuclei while both sharing the same centroids. Having said that, the main output of this step is the cell masks.

#### 4.4.4. Feature extraction and output products

Following the segmentation of probability maps, we create two sets of image masks for each detected cell. One is associated with the segmented cells and the other with the cell’s nuclei. For each detected pair of cell/nucleus, the pipeline uses associated masks to compute mean pixel intensities inside the area of the cell/nucleus across all available IMC channels. Next, image moments are estimated for each set of cell/nucleus to find corresponding areas. Finally, the pipeline exports a table where each row represents a cell with all extracted parameters as feature columns. In addition, the code exports a mask images of all detected cells/nuclei for the followed-up single-cell analysis.

#### 4.4.5. Performance

The performance of a segmentation algorithm depends on several factors. For instance, the tissue preparation method (e.g., frozen vs. Formalin Fixed Paraffin Embedded), its type and thickness and the scanning resolution all affect the accuracy and performance of a segmentation algorithm. These are apart from other factors such as fine-tuning the input parameters of an algorithm or hyper-parameters of neural-net based models. Therefore, the best way to assess the performance of an algorithm is to test it on specific data that is subject to the segmentation analysis. For the current IMC pipeline, we measure the conventional pixel-wise F1 and object-wise F1 scores. For this, we selected 10 IMC image tiles regions ( 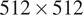 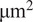 ) with varying morphology and cell counts, and performed a manual annotation of all the cell nuclei. This resulted in an average of 829 segmented cells per tile. Comparing the automatic cell segmentation with these manual annotations provided a mean 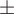 standard deviation pixel-wise F1 score of 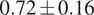 and object-wise F1 score of 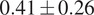 . Finally, as a preliminary test, we run the pipeline on a sample of breast cancer patient-derived tumor xenograft (PDTX) that was previously analyzed with mass cytometry (MC). Results as analyzed using the current IMC pipeline, successfully reveal the spatial distribution of cell phenotypes in xenografts as observed with MC data. The study finds that centroids of each cell cluster computed per PDX model on the MC training data shows a high correlation ( 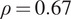 ) with the corresponding centroid following cell segmentation and classification using the IMC pipeline^(^^12^^)^.

### 4.5. STPT tissue segmentation pipeline

While IMC is suitable for cell segmentation, the STPT modality is particularly suitable for visualizing the various tissue structures, such as the stroma and vasculature. To be able to quantify these structures, several tool have been developed.

#### 4.5.1. Section resampling

The STPT images are loaded at the level corresponding to a 4 times downsampling. They are subsequently resampled to a pixel size of half the slice spacing, that is, 7.5 μm, while applying the *x*- and *y*-translations which were calculated during stitching and registration.

#### 4.5.2. Upsampling

In the current setup, 15 μm sections are acquired. This may cause some discontinuity between sections for oblique tissue structures, as is the case with blood vessels. To resolve this we can apply an up-sampling based on an intensity-based deformable registration. With a pixel size set to half the slice spacing, a linear interpolation is performed between each pair of neighboring sections along the direction calculated by the registration. In this way, we generate interpolated slices throughout the volume. This increases the out of plane resolution and improves the overall resolution when converting to a volume with an isotropic voxel spacing, which is required for some of the measurements.

#### 4.5.3. Segmentation

Segmenting the tissue structures is done using a manually defined threshold value resulting in a binary segmentation. This is followed by a smoothing, which is performed separately for the in-plane and out-of-plane direction due to differences in the signal to noise ratio between the two. Finally, a connected component filter is applied to remove the small structures, which can be considered noise.

#### 4.5.4. Quantification

Once the tissue is segmented, several structural parameters are extracted. These include the tissue ratios, the tissue thickness, the fractal dimension, and the connectivity density.

### 4.6. Data federation

While STPT process, the whole sample sequentially, other modalities like Axioscan or IMC work over single slices. This implies that slice-to-slice registration may not be possible for these modalities, and in order to recover the 3D position of data from them it is required to relate them to the STPT data.

As discussed previously, physical slices are recovered from the STPT microscope in random order and deposited onto glass slides that then go through the Axioscan. The first step then in our multimodal registration is to find the best Axioscan-STPT pairs, so that we can assign a *Z* coordinate (i.e., depth in the sample) to each Axiocan slide image.

A comprehensive review of multi-modality image registration methods is out of scope here. In general, most methods can be divided into classic methods^(^^41^^)^ and methods based on deep learning^(^^42^^)^. Classical methods rely on detecting a set of common features on the images that need to be registered in order to find the best feature matches and determine a transformation. Methods based on deep learning techniques are varied across the literature but their use is limited in our case due to the varied image modalities and the need of training sets. The main problem with multi-modal images is that of detecting and matching features in images that look different because they are observed by different instruments that highlight different features of the tissue and image different parts of the sample (e.g., STPT signal comes from a thin section of a few microns while Axioscan images the whole 15 micron slice). Other issues that we need to have into account include:

- The slices can suffer mechanical deformation when being deposited onto the glass slides, leading to imperfect matches between Axioscan and STPT.
- The orientation of the slice on the glass slide is random, leading to possible left/right and top/bottom inversions.
- Not all Axioscan images may have a good STPT match. Because the first STPT image will be taken at some depth into the sample, the microtome may cut above this layer, leading to an orphan physical slice.

Fiducial beads provide an ideal set of common features that are detected in all the images independently on which modality they have been acquired. Even if the characteristics of the beads like size or illumination vary across the different modalities, their center can be calculated with high level of accuracy (best than a quarter of a pixel).

The problem is then to find the best correspondence between the catalogue of 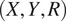 for a given Axioscan image and the 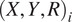 for all the STPT slices.

Firstly we will search for a more complex transformation than the simple displacements used when registering STPT slices. We settle for an affine transformation:

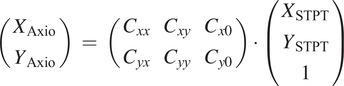



**Figure 19.**
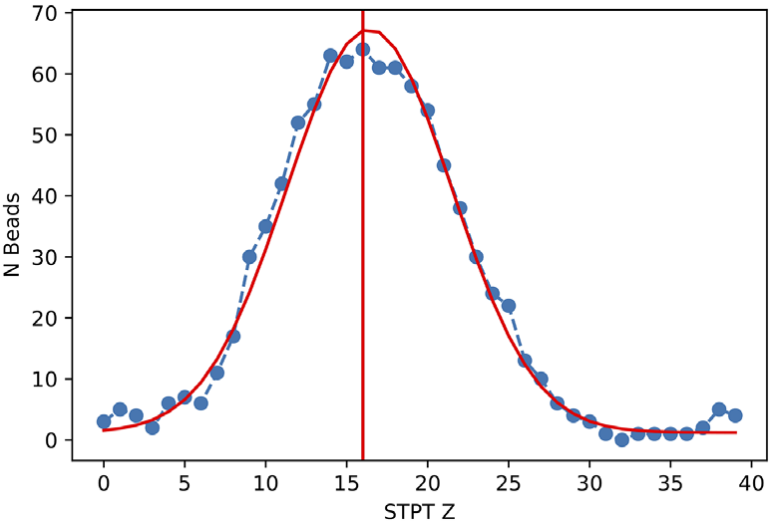
Number of common beads between an Axioscan and all the STPT slices taken from the parent sample cube. The red line is the best Gaussian fit to the evolution of 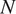 with 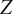 , and the vertical line marks the predicted 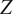 for 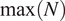 .

Secondly, we jumpstart the algorithm with a coarse intensity match between a 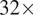 downsampled Axioscan image and the median STPT image for all the slices, also downsampled. This will give us the left/right and top/bottom relative orientations, and an initial estimation of the matrix of coefficients 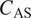 . For each STPT slice, we refine this matrix by finding the coefficients that maximize the number 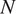 of bead matches 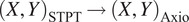 within 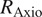 . 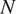 should increase monotonically with STPT Z until it reaches a maximum for the most similar slice, and then onwards it should decrease as we move away from this slice. In reality (Figure 19), 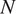 is a noisy function of 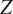 , and so we fit a smooth function (a simple Gaussian) and find the 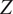 value closest to the function maximum.

**Figure 20.**
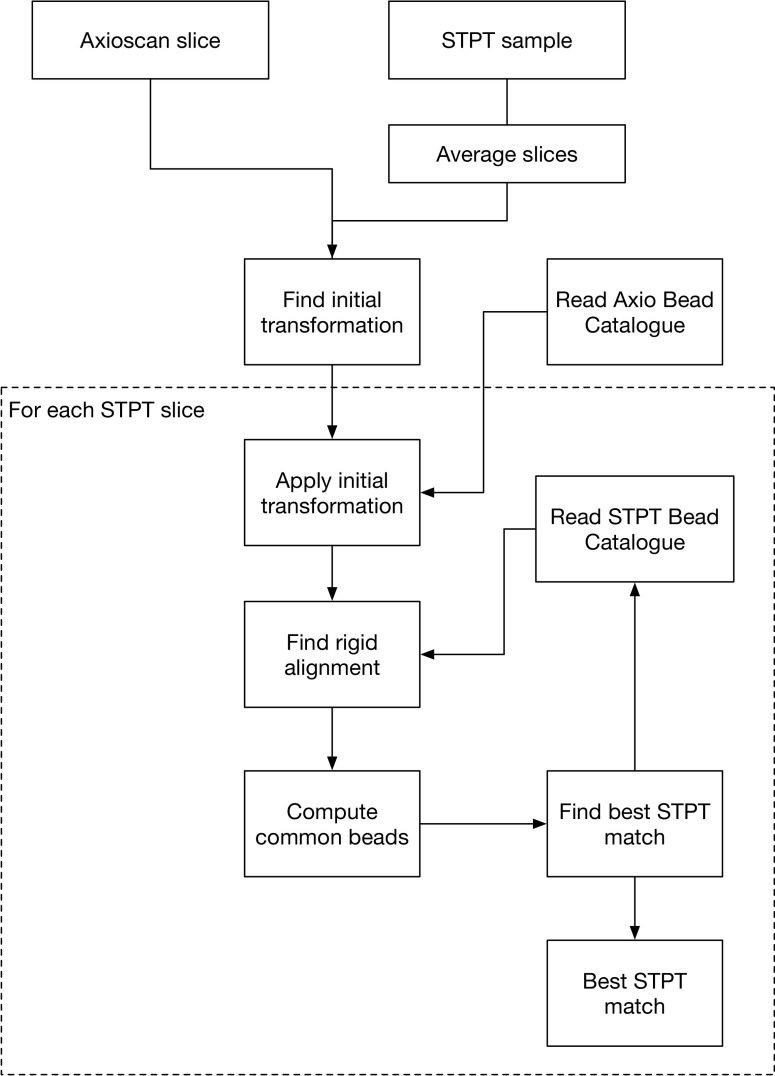
Axioscan to STPT slide matching algorithm.

The result from this algorithm is the best matching STPT slice along with the corresponding affine transform between both pixel coordinates. The algorithm is summarized below and in Figure 20.

Once the slices are scanned they are given an identifier that simplifies further registration. Some of these slices will go to IMC, and these need to be registered back to STPT too. We use Axioscan as a sort of man-in-the-middle between IMC and STPT. Because once the slices are deposited onto the glass slides they are more or less stable,^2^ IMC to Axioscan registration is quite easy, despite the fact that normally, due to the time cost involved, the portion of slice sampled with IMC tends to be small (and therefore to have few beads). Beads are detected on the IMC data in a similar manner as detailed before, and because we know which Axioscan slice corresponds to which IMC, we just need to find the nearest coordinates matches between bead positions 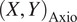 to 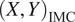 and obtain the coefficients 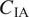 associated with this transformation.

**Figure 21.**
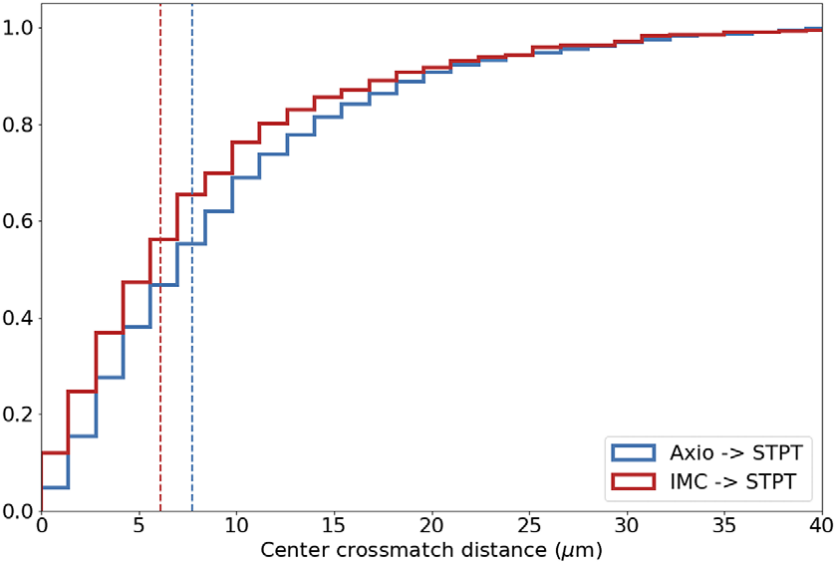
Cumulative distribution for the registration errors as measured through the reprojected center coordinates.

With this new matrix, we can compound the transformation

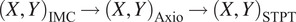

and obtain a first estimation of 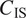 ; with this first crossmatch between IMC and STPT through this route, we can refine the transformation and refine the coefficients of 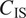 . We estimate the error of this procedure through the Cartesian distances between STPT beads and the respective reprojected modalities. The results are summarized in Figure 21; median error for the IMC to STPT registration is of 6 μm, while this figure is of 7 μm for Axioscan to STPT. These differences are mediated by the number of visible beads and the native pixel size in each modality.

A model with 6 degrees of freedom like the one we propose here may appear to be simple, but it works to sub-cellular precisions over samples with sizes of 1 cm 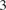 . These affine transforms have the added advantage that are easy to encode, all the coefficients have physical interpretation (rotation, scale, shear) and once we have the 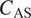 , 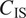 and 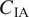 matrixes, it is possible to reproject segmentation catalogues and masks from information-dense modalities like IMC over time efficient imagers like STPT.

As with STPT slice-to-slice registration, the algorithm for bead detection and profiling works well when recovering bead centers down to very low S/N, but the accuracy of the registration depends strongly on the number of available beads. As can be seen in Figure 19, this is unlikely to be a difficulty for STPT-Axio registration, but it will be a bigger issue when dealing with IMC data; as discussed earlier, the field-of-view in this modality tends to be small, and therefore only beads close to the sample are likely to be captured. But since physical deformations between Axio and IMC are negligible, in case of critically low number of beads, the transformation matrix between these modalities could be simplified without a significant loss of precision, and the compounded with the better determined Axioscan to STPT transformation in order to obtain an indirect IMC to STPT registration.

An underlying assumption for this registration is that the biological sample and the beads behave in a similar way. As can be seen in Figures 10 and 12, the beads surround the sample, but there are no fiducial marks inside the sample itself. One of the implications of this is that the errors in Figure 21 are likely a higher envelope of the real registration errors. The STPT mosaic is built by intensity-matching tile overlaps. Because as we move away from the sample the information in these overlaps decreases, the outer areas of the sample, where a large fraction of the beads sit, will be relatively worse stitched (as often we will need to rely on the default microscope displacements) than those near the sample, and therefore the former may dominate the registration error budget.

## Data Availability

IMAXT aims to produce periodic data releases, including processed imaging data, segmentation catalogues, federated datasets, masks used for training segmentation neural networks, and so on. We also plan to open our infrastructure to external users to perform their analysis. Information on these data releases and how to access the data can be accessed from https://imaxt.ast.cam.ac.uk/release. All the code to perform stitching, registration, and segmentation as well as additional tools is available in the IMAXT GitHub organization at https://github.com/IMAXT/ under a GNU General Public License version 3 (GPLv3). In particular, the STPT stitching and registration pipeline is available from https://github.com/IMAXT/stpt-mosaic-pipeline and the IMC nuclear channel segmentation pipeline in https://github.com/IMAXT/imc-segmentation-pipeline. Additional information on our infrastructure, software tools, data model, and applications is available from https://imaxt.github.io.

## Footnotes

1 We currently use 270 images that are split and augmented to 600 batches of 128 annotated images each.

2 Prior to IMC, there is a high-temperature drying process that we have found does not deform the sample slices appreciably.

## CRUK IMAXT Grand Challenge Consortium

H. Raza Ali, M. Al Sad, S. Alon, Samuel Aparicio, G. Battistoni, S. Balasubramanian, R. Becker, Bernd Bodenmiller, E. S. Boyden, D. Bressan, A. Bruna, B. Marcel, Carlos Caldas, M. Callari, I. G. Cannell, H. Casbolt, N. Chornay, Y. Cui, A. Dariush, K. Dinh, A. Emenari, Y. Eyal-Lubling, J. Fan, E. Fisher, E. A. González-Solares, C. González-Fernández, D. Goodwin, W. Greenwood, F. Grimaldi, G. J. Hannon, O. Harris, S. Harris, C. Jauset, J. A. Joyce, E. D. Karagiannis, T. Kovaevi, L. Kuett, R. Kunes, A. Küpcü Yolda, D. Lai, E. Laks, H. Lee, M. Lee, G. Lerda, Y. Li, J. C. Marioni, A. McPherson, N. Millar, C. M. Mulvey, F. Nugent, C. H. O’Flanagan, M. Paez-Ribes, I. Pearsall, F. Qosaj, A. J. Roth, Oscar M. Rueda, T. Ruiz, K. Sawicka, L. A. Sepúlveda, S. P. Shah, A. Shea, A. Sinha, A. Smith, S. Tavaré, S. Tietscher, I. Vázquez-García, S. L. Vogl, N. A. Walton, A. T. Wassie, S. S. Watson, T. Whitmarsh, S. A. Wild, E. Williams, J. Windhager, C. Xia, P. Zheng, and X. Zhuang.
